# Brain Microdialysate Monoamines in Relation to Circadian Rhythms, Sleep, and Sleep Deprivation – a Systematic Review, Network Meta-analysis, and New Primary Data

**DOI:** 10.5334/jcr.174

**Published:** 2019-01-14

**Authors:** Julia M. L. Menon, Christ Nolten, E. J. Marijke Achterberg, Ruud N. J. M. A. Joosten, Maurice Dematteis, Matthijs G. P. Feenstra, W. H. (Pim) Drinkenburg, Cathalijn H. C. Leenaars

**Affiliations:** 1 Radboud Institute for Health Sciences, Radboud University Medical Centre, Nijmegen, NL; 2 Janssen Research and Development, a division of Janssen Pharmaceutica N.V, Beerse, BE; 3 Department of Animals in Science and Society – Human-Animal Relationship, Utrecht University, Utrecht, NL; 4 Netherlands Institute for Neuroscience (NIN), An Institute of the Royal Netherlands Academy of Arts and Sciences, Meibergdreef 47, 1105BA Amsterdam, NL; 5 Grenobles Alpes University Hospital and Grenoble Alpes University, Faculty of Medicine, FR; 6 Institute for Laboratory Animal Science, Hannover Medical School, Hannover, DE

**Keywords:** Systematic review, network meta-analysis, microdialysis, monoamines, circadian rhythm, sleep deprivation

## Abstract

Disruption of the monoaminergic system, e.g. by sleep deprivation (SD), seems to promote certain diseases. Assessment of monoamine levels over the circadian cycle, during different sleep stages and during SD is instrumental to understand the molecular dynamics during and after SD. To provide a complete overview of all available evidence, we performed a systematic review. A comprehensive search was performed for microdialysis and certain monoamines (dopamine, serotonin, noradrenaline, adrenaline), certain monoamine metabolites (3,4-dihydroxyphenylacetic acid (DOPAC), 5-hydroxyindoleacetic acid (5-HIAA)) and a precursor (5-hydroxytryptophan (5-HTP)) in PubMed and EMBASE. After screening of the search results by two independent reviewers, 94 publications were included. All results were tabulated and described qualitatively. Network-meta analyses (NMAs) were performed to compare noradrenaline and serotonin concentrations between sleep stages. We further present experimental monoamine data from the medial prefrontal cortical (mPFC). Monoamine levels varied with brain region and circadian cycle. During sleep, monoamine levels generally decreased compared to wake. These qualitative observations were supported by the NMAs: noradrenaline and serotonin levels decreased from wakefulness to slow wave sleep and decreased further during Rapid Eye Movement sleep. In contrast, monoamine levels generally increased during SD, and sometimes remained high even during subsequent recovery. Decreases during or after SD were only reported for serotonin. In our experiment, SD did not affect any of the mPFC monoamine levels. Concluding, monoamine levels vary over the light-dark cycle and between sleep stages. SD modifies the patterns, with effects sometimes lasting beyond the SD period.

## Introduction

Circadian rhythms (CRs) and sleep are influenced by multiple external (e.g. light) and internal (e.g. accumulation of hypnogenic substances) factors [1]. Our relationship to light cues and rhythms has become disturbed in our industrialized 24/7 society. This resulted in increased prevalences of CR desynchrony and impaired sleep, which in turn severely impact health [2].

Unusual working hours can result in low sleep quality, consumption of stimulants and/or hypnotics, and stress. These factors could all contribute to sleep-deprivation associated disorders (e.g. insomnia and mental illnesses) [3,4,5,6,7].

Sleep deprivation (SD) induces severe cognitive impairments such as loss of attention, increased reaction times, impaired multitasking and planning, slurred speech, impaired memory, and poor emotion regulation [8,9,10]. Mainly the loss of attention affects safety e.g. in driving [11] and dangerous work [12,13]. Furthermore, SD and CR disruption are associated with diseases such as obesity, sleep apnoea, diabetes, cardiovascular diseases, and depression [2,14].

While the behavioural impact of SD is well-known, our knowledge of the responsible underlying neurological mechanism is still limited. Sleep is embedded in a complex network of interconnected brain regions using multiple neurotransmitters and neuromodulators, within which the monoaminergic pathways seem responsible for sleep-wake modulation [15,16]. In addition, monoamines play a role in certain cognitive processes that are disrupted by SD [17,18,19]. Therefore, it is important to provide a complete overview of how the release of these monoamines is related to circadian rhythms and how it is affected by sleep deprivation.

A well-established way to study the release of neurotransmitters is to measure them with microdialysis. Microdialysis is a versatile technique to study the extracellular space *in vivo*, based on the simple principle of diffusion [20]. A probe with a semi-permeable membrane is placed in a region of interest. When the probe is perfused continuously with an isotonic solution, substances in the extracellular space (e.g. neurotransmitters and neuromodulators) will diffuse through the membrane into the perfusion fluid, which is collected for analysis. The concentrations in the perfusate reflect neuronal release and are dependent on neuronal activity [21,22]. Microdialysis allows for measurements on a minutes-hours timescale for several compounds simultaneously [23,24].

Because monoamines seem to be involved in many of the functions disrupted by SD, we performed a systematic review of *in vivo* extracellular concentration of several monoamines [i.e. dopamine (DA), serotonin (5-HT), noradrenaline (NA), adrenaline (ADRE)) and related compounds (i.e. 3,4-dihydroxyphenylacetic acid (DOPAC), 5-hydroxytryptophan (5-HTP), and 5-hydroxyindoleacetic acid (5-HIAA)) in relation to 1.) CRs, 2.) naturally occurring sleep stages, and 3.) SD. We excluded the monoamine histamine from this review, because we included it in a comparable review on amino acids [25], aligned with our primary data.

Systematic reviews (SRs) provide all available evidence on a subject in a complete and organized manner (i.e. transparent and reproducible methodology) [26]. Even though numerous excellent narrative reviews exist on microdialysis (e.g. [27]), SRs combining microdialysis and monoamine measurements are scarce. The two examples we are aware of describe the enhancement of monoamine levels by ethanol administration [28] and serotonin neurotransmission after administration of selective serotonin reuptake inhibitors [29]. Other SRs on the microdialysis technique addressed measurements of amino acids [30], acetylcholine [31], and adenosine [32].

The research questions for this SR were to determine whether and how monoamine concentrations are influenced by 1) CRs; 2) naturally occurring sleep-wake stages, and 3) SD. We provide qualitative descriptions of the overall trends and quantitative comparison of monoamine levels between wake-sleep stages.

In addition to our SR, we present data from an unpublished study of the medial prefrontal cortex (mPFC) before, during and after SD. These data are the first published on several monoamines during SD in the mPFC. Our microdialysis experiment was designed in line with our preceding behavioural work, showing that SD affects certain but not all mPFC related cognitive tasks in rats [33,34].

## Material and Methods

In this method section we first describe our systematic literature review, and then our experimental data collection. We wrote a protocol for the review before starting the selection of publications. The protocol was posted to the SyRCLE website (www.SYRCLE.nl) on 20 October 2017 [35].

### Systematic review

#### Search and selection

Our extensive search strategy consisted of three components: “circadian rhythm, sleep, and sleep deprivation”, “neurotransmitters and metabolites” and “microdialysis”. The full search strategy is provided in our protocol [35]. We searched PubMed and EMBASE on 18 September 2017. Duplicates and triplicates were manually removed.

Screening was conducted in EROS (Early Review Organising Software; Institute of Clinical Effectiveness and Health Policy, Buenos Aires, Argentina) by two independent reviewers (JMLM and CHCL for title abstract screening, and JMLM, CHCL or EJMA for full text screening). Discrepancies were discussed among reviewers until consensus was reached. We excluded publications on other techniques than microdialysis, extracerebral and *in vitro* microdialysis, and other substances than dopamine, noradrenaline, adrenaline, serotonin, 5-HTP, DOPAC and 5-HIAA. During full text screening we further excluded publications not describing sleep-related conditions and/or prolonged baseline for CRs. Sleep-related conditions comprised SD, naturally occurring sleep-wake stages and models for sleep disorders. Prolonged baseline was defined as “an uninterrupted and undisturbed period of at least 6h within which one light-dark transfer occurs”. Publications were included regardless of species, year of publication, language and type of experiment. We only included peer-reviewed publications.

We excluded publications on CRs shifts, and, deviating from our protocol, those on sleep disordered animals (e.g. [36]) because of the limited number of studies retrieved by our searches. Further deviating from our protocol, we retrieved additional references by checking the reference lists of all reviews encountered during full text screening. Listed references with “release” or words starting with “dialys*” in their title and those otherwise deemed relevant by the review authors were retrieved for screening.

#### Data extraction and quality assessment

Data were extracted on study design (e.g. independent or dependent groups), animal model (e.g. strain, sex), microdialysis technique (e.g. flow rate, perfusion medium) and outcome measurements (type of monoamine, concentration or % of baseline). From one publication, we extracted as much as possible without knowledge of Japanese [37]. All other included publications were in English.

Microdialysis experimental methods are heterogeneous due to the versatility of the technique. For example, separate experimental groups can be used for different interventions or brain regions, but within-subject cross-over designs and simultaneous measurements in several brain regions are also common. When publications described separate experimental groups, the groups were treated as independent experiments, from hereon called “studies” (indicated by upper case letters in the reference_ID, in contrast to publications from the same authors and same year, which are indicated by lower case letters). If measurements were simultaneously performed in several brain regions within the same animals (k = 34 studies), the brain regions were treated as the experimental unit (instead of the animal), also called “studies”. We only included “studies” meeting our inclusion criteria.

For publications using seals (k = 4), each seal was treated as an independent observation, unless only pooled data were presented. This was deemed necessary because of the low number of animals included per study, and the high diversity in the probe numbers and locations per animal.

Extracted data were tabulated in Excel. Outcomes were sorted by monoamine (dopamine, serotonin, noradrenaline, adrenaline, DOPAC, and 5-HIAA) and review question (CRs, sleep, and SD; results section 3.3–3.5). Study characteristics were tabulated per review question (appendix 1–3).

As a crude measure of study (reporting) quality, we calculated percentages of reported study characteristics for our sample of studies. We created a list of characteristics based on the SYRCLE risk of bias tool [38], adapted it to sleep studies as partially described before [39], and added specific elements for microdialysis studies, as described in appendix 4.

### Network meta-analysis and meta-analysis

Data were copied from texts and tables, or, if no numerical data were provided, extracted from figures with a graphical ruler (Universal graphic ruler, v3.8.6498). Concentrations were converted into nmol/L (nM) if necessary.

In our protocol we only specified that a meta-analysis would be designed and performed if at least 2 included articles measured the same monoamine in the same condition. As sufficient data were available on serotonin and noradrenaline during the naturally occurring sleep stages (Slow Wave Sleep (SWS) and Rapid Eye Movement (REM) sleep) and wakefulness, we decided to conduct two network meta-analyses (NMAs). Five studies provided concentrations for SWS and wake only, because the REM sleep episodes were too short to collect a full dialysate sample, or because REM was not observed.

If two values were given for the same stage, we conservatively assumed a correlation of 1 between them and included the mathematical average in the NMAs [40]. If the number of animals was only provided as a range throughout the experiment, the median was used for the NMAs.

Seal studies were excluded from the NMAs because of their previously described complex designs, besides the uniqueness of seals’ sleep patterns comprising unilateral sleep. The study of Zeitzer *et al* [41] was excluded from the NMAs because of its low power; repeated measurements were performed over one night in one human. The study of Bellesi *et al* [42] was excluded as we could not calculate the actual monoamine concentrations from the provided percentages.

Analyses were conducted in R, version 3.4.3 (2017-11-30) – “Kite-Eating Tree” using the netmeta package. We used the netmeta function with random effect models and standardised mean differences.

To verify the NMA results, we conducted four regular meta-analyses (MAs), using the metacont function and forest plots from the meta and metafor packages, hakn = TRUE.

### Experiment: Prefrontal cortex primary data before, during and after SD in rats

Methods for this experiment have been described previously [43]. Shortly, 11 Wistar rats were implanted with custom-made concentric microdialysis probes (4mm membrane length) in the medial prefrontal cortex (mPFC) at an angle of 12° (AP+3.0mm; L ± 1.8; V–5.5; relative to Bregma). Rats recovered for approximately one-week post-surgery before the start of the experiment. They were connected to the microdialysis tubing and placed into separate compartments of SD devices. Artificial cerebrospinal fluid (145 mmol/l NaCl, 1.2 mmol/l CaCl_2_, 2.7 mmol/l KCl, 1.0 mmol/l MgCl_2_) was perfused through the probe at a flow rate of 3 μl/min. Rats habituated to the experimental set up for 12h. After this period, 24h of baseline measurements were followed by 12h of SD during the light phase (modelling a sleepless night in humans) and 16h of recovery. The experiment was approved by the experimental animal committee of the Royal Netherlands Academy of Arts and Sciences and performed in accordance with European guidelines and Dutch legislation (Wet Op de Dierproeven, 1996).

Dialysates were collected in one-hour samples (180 μL) in 300μL plastic vials (7431100, Aurora Borealis) placed in a refrigerated fraction collector (6°C; CMA 470, Aurora Borealis). Samples were transferred to ice and split into 8 fractions. After the experiment, fractions were stored at –80°C. The fractions used for monoamine measurements (20 μL) were transported on dry ice from Amsterdam to Beerse. Monoamines were determined by Janssen Pharmaceutica, Research & Development (Beerse, Belgium), department of Neuroscience Systems Biology. Their standard protocol comprises HPLC-FD following subsequent derivatization with benzylamine and 1,2-diphenylethylenediamine, as described before by Fujino *et al* [44].

To prevent outliers affecting our results, we selected a non-parametric approach for data analysis. Monoamine median and interquartile ranges were calculated for each stage (light phase, dark phase, SD, and recovery) in Excel. Friedman’s ANOVA’s were performed in SPSS version 22 for each monoamine separately. If the Friedman’s test was significant, post-hoc Wilcoxon tests were performed to compare baseline light with baseline dark, and SD and recovery with the corresponding baseline period.

A first paper on the validation of our method for SD showed dialysate corticosterone concentrations [43]; a second paper showed adenosine concentrations from the same experiment [32]. A paper on amino acids is in progress.

## Results

In this section, we present the results of our systematic review, followed by our experimental data.

**Figure 1:**
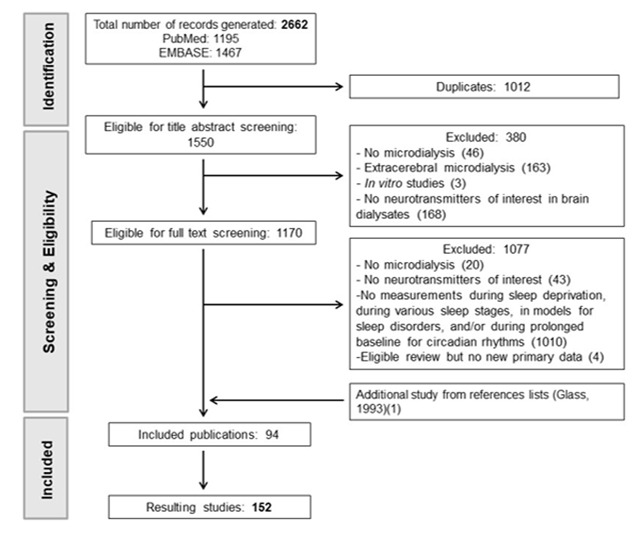
Flow scheme of screening process.

**Figure 2:**
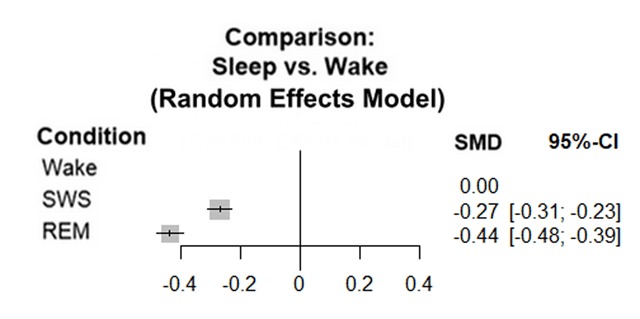
Network meta-analysis comparing serotonin levels during wakefulness, SWS and REM sleep. This plot summarises the results of 26 studies; 19 had data for each stage, 7 had data only for wakefulness and SWS. For the overall effect, p < 0.0001. The analysis shows significant heterogeneity; Τ² = 0.0059; I² = 98.4%. Abbreviations: SWS: Slow Wave Sleep; REM: Rapid Eye Movement sleep.

**Figure 3:**
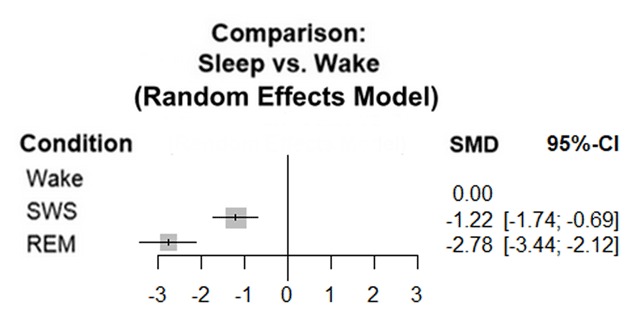
Network meta-analysis of noradrenaline levels during wakefulness, SWS and REM sleep. This plot summarises the results of 13 studies; 8 had data for each stage, 5 had data only for wakefulness and SWS. For the overall effect p < 0.0001. The analysis shows significant heterogeneity; Τ² = 0.7835; I² = 99.5%. Abbreviations: SWS: Slow Wave Sleep; REM: Rapid Eye Movement sleep.

For the review, we start with a description of the publications retrieved from the search and the selection process (Figure 1), we then describe the study characteristic of the included publications, and we finish with qualitative descriptions of the monoamine concentrations for CR (Tables 1, 2, 3, 4, 5, 6), naturally occurring sleep stages (Tables 7, 8, 9, 10, 11), and SD (Tables 12, 13, 14, 15, 16, 17). The section on sleep stages comprises the network meta-analyses of serotonin and noradrenaline (Figures 2–3).

**Table 1:** Circadian rhythms in dopamine levels.

Dopamine-Circadian Rhythms					
Reference_ID	Animals	L/D Cycle	Duration	Brain Region	Dopamine Levels

Dugovic et al (2009) [54]	Rats	6h–18h	6h	Prefrontal Cortex	Higher during DP, lower during DP
Barbier et al (2007) [55]	Rats	6h–18h	20h	Prefrontal Cortex	Fairly stable
Nakayama et al (1993) [56]	Rats	8h–20h	24h	Medial Prefrontal Cortex	Higher during DP, lower during LP No effect of extra 12h DP
Robinson et al (1991) [57]	Sheep	Natural cycle	20h	Preoptic Area	Stable during DP, higher during LP
Alfinito et al (2009) [58]	Rats	12:12	12h30	Preoptic Area	Stable
Smith et al (1992) [46]	Rats	7h–19h	18h	Striatum	Higher during DP, lower during LP
Castaneda et al (2004) A1 [59]	Rats	20h–8h	30h	Striatum	Lower during DP, higher during LP
Castaneda et al (2004) B1 [59]	Rats	6h DP–24h LP	30h	Striatum	Higher at DP onset, then decrease and reach its lowest during LP
Hood et al (2010) [60]	Rats	8h–20h	24h	Striatum	Higher during DP, lower during LP
Sano et al (1992) A [47]	Rats, young animals	6h–18h	24h	Striatum	Higher during DP, lower during LP
Sano et al (1992) B [47]	Rats, old animals	6h–18h	24h	Striatum	Same pattern but levels are lower
Sano et al (1992) C [47]	Rats, enriched milieu	6h–18h	24h	Striatum	Stable
Sano et al (1992) D [47]	Rats, isolated	6h–18h	24h	Striatum	Stable
Decker et al (2005) [53]	Rats	7h–19h	48h	Striatum	A few spikes but mean is stable between DP and LP
De Marquez-Pardo et al (2000) [52]	Rats	8h–20h	24h	Neostriatum	Higher during DP, lower during LP
Ferris et al (2014) A [48]	Rats	ZT0–ZT12	36h	Caudate Putamen	Higher during DP, lower during LP
Ferris et al (2014) B [48]	Mice	ZT0–ZT12	38h	Caudate Putamen	Peak at DP onset, higher during DP, lower during LP
Ferris et al (2014) C [48]	Mice (DAT KO)	ZT0–ZT12	38h	Caudate Putamen	Stable
Paulson et al (1994) 1 [61]	Rats	6h–20h	20h	Caudate Nucleus	Higher during DP (double the NAC levels), lower during LP
Paulson et al (1996) 1 [62]	Rats	6h–20h	18h20	Caudate Nucleus	Higher during DP, lower during LP
Murillo-Rodriguez et al (2013) [63]	Rats	7h–19h	6h	Nucleus Accumbens	Stable
Paulson et al (1994) 2 [61]	Rats	6h–20h	20h	Nucleus Accumbens	Stable
Paulson et al (1996) 2 [62]	Rats	6h–20h	18h20	Nucleus Accumbens	Higher during DP, lower during LP
Castaneda et al (2004) A2 [59]	Rats	20h–8h	30h	Nucleus Accumbens	Lower during DP, higher during LP
Castaneda et al (2004) B2 [59]	Rats	6h DP–24h LP	30h	Nucleus Accumbens	Higher during DP, lower during LP
Verhagen et al (2009) [64]	Rats	2h–14h	36h	Lateral to Nucleus Accumbens Shell	Higher during DP, lower during LP
Fetissov et al (2000) 1 [65]	Rats	6h–18h	24h	Lateral Hypothalamus	Peak at DP onset, then start to decrease after 2h. Stay stable during LP
Fetissov et al (2000) 2 [65]	Rats	6h–18h	24h	Ventromedial Hypothalamus	Gradually decrease
Izumo et al (2012) [66]	Rats	7h–19h	15h	Central Nucleus of the Amygdala	Higher during DP, lower during LP (wide error bars)

Each row represents one study (i.e. an experimental group within a publication) and a qualitative description of the results. Fluctuations are described as “higher” and “lower” disregarding actual magnitude of changes. Rows are sorted by brain region. Lower case letters indicate separate publications from the same authors in the same year; upper cases letters represent separate groups within publications; numbers represent separate brain regions within animals. Abbreviations: L/D cycle: Light-Dark Cycle; LP: Light Phase; DP: Dark Phase; DAT KO: Dopamine Transporter Knock Out; ZT: Zeitgeber.

**Table 2:** Circadian rhythms in DOPAC levels.

DOPAC- Circadian Rhythms					
Reference_ID	Animals	L/D Cycle	Duration	Brain Region	DOPAC Levels

Ferris et al (2014) A [48]	Rats	ZT0–ZT12	36h	Caudate Putamen	Higher during DP, lower during LP
Paulson et al (1994) [61]	Rats	6h–20h	20h	Caudate Nucleus	Higher during DP, lower/stable during LP
Paulson et al (1996) [62]	Rats	6h–20h	18h20	Caudate Nucleus	Higher during DP, lower during LP
Castaneda et al (2004) A1 [59]	Rats	20h–8h	30h	Striatum	Higher during DP, lower during LP
Castaneda et al (2004) B1 [59]	Rats	6h DP–24h LP	30h	Striatum	Higher during DP, lower during LP
Hucke et al (1998) A [51]	Rats, nulliparous	6h–18h	8h	Striatum	Higher during DP, stable during LP
Hucke et al (1998) B [51]	Rats, primiparous	6h–18h	8h	Striatum	Higher during DP, stable during LP
Sano et al (1992) A [47]	Rats, young animals	6h–18h	24h	Striatum	Higher during DP, lower during LP. Highest values mid DP, lowest values mid LP
Sano et al (1992) B [47]	Rats, old animals	6h–18h	24h	Striatum	Smaller variation and level of DOPAC than young group
Sano et al (1992) C [47]	Rats, isolated	6h–18h	24h	Striatum	Stable
Sano et al (1992) D [47]	Rats, enriched environment	6h–18h	24h	Striatum	Higher levels than isolated, higher during DP, lower during LP
Smith et al (1992) [46]	Rats	7h–19h	18h	Striatum	Highest during LP, decrease gradually during the entire duration, reach lowest during DP
De Marquez-Prado et al (2000) [52]	Rats	8h–20h	24h	Neostriatum	Decrease during the entire duration (start at DP)
Castaneda et al (2004) A2 [59]	Rats	20h–8h	30h	Nucleus Accumbens	Higher during DP, lower during LP
Castaneda et al (2004) B2 [59]	Rats	6h DP–24h LP	30h	Nucleus Accumbens	Higher during DP, lower during LP.
Paulson et al (1994) [61]	Rats	6h–20h	20h	Nucleus Accumbens	Increase during LP to be the highest at DP onset. Stay stable during DP, lower during LP
Paulson et al (1996) [62]	Rats	6h–20h	18h20	Nucleus Accumbens	Higher during DP, lower during LP
Verhagen et al (2009) [64]	Rats	2h–14h	36h	Lateral to Nucleus Accumbens Shell	Higher during DP, highest at the end of DP Lowest level mid-LP. High range of fluctuation.
Nakayama et al (1993) [56]	Rats	8h–20h	24h	Medial Prefrontal Cortex	Peak at DP onset and about 3/4 DP, decrease strongly between the 2 peaks. Decrease during LP
Luo et al (2014) [67]	Rats	?	24h	SCN	Higher during DP

Each row represents one study (i.e. an experimental group within a publication) and a qualitative description of the results. Fluctuations are described as “higher” and “lower” disregarding actual magnitudes of changes. Rows are sorted by brain region. Lower case letters indicate separate publications from the same authors in the same year; upper cases letters represent separate groups within publications; numbers represent separate brain regions within animals. Abbreviations: DOPAC: 3,4-Dihydroxyphenylacetic acid; L/D Cycle: Light-Dark Cycle; LP: Light Phase; DP: Dark Phase; SCN: Suprachiasmatic Nucleus; ZT: Zeitgeber.

**Table 3:** Circadian rhythms in serotonin levels.

Serotonin-Circadian Rhythms					

Reference_ID	Animals	L/D Cycle	Duration	Brain Region	Serotonin Levels

Huang et al (2008) [68]	Rats	6h–18h	72h	Pineal Gland	Peak at DP onset, then decrease at its lowest, before increasing again before LP. Stable during LP.
Sun et al (2002) [69]	Rats	11h–1h	312h	Pineal Gland	Increase strongly at DP onset, then decrease gradually, increase at the end. Stable during LP.
Sun et al (2003) [70]	Rats	11h–1h	132h	Pineal Gland	Peak at DP onset, gradual decrease during the rest of DP. Increase during LP.
Azekawa et al (1991) [71]	Rats	7h–19h	24h	Pineal Gland	Peak after DP onset followed by strong decrease until mid DP. Then increase until LP onset. Lower during LP
Liu et al (2005) [72]	Rats	11h–23h	72h	Pineal Gland	Peak 1h after DP onset, and 3h before LP. Nadir is seen at LP beginning, followed by a gradual increase until DP onset.
Liu et al (2006) A [73]	Rats (LEW)	6h–18h	120h	Pineal Gland	Higher after DP onset, followed by a sharp decrease until the end of DP. Levels return to baseline level and stay stable during LP
Liu et al (2006) B [73]	Rats (SD)	6h–18h	24h	Pineal Gland	Higher after DP onset but shifted compared to LEW followed by a strong decrease until the end of DP. Return to baseline level and stay stable during LP
Liu et al (2006) C [73]	Rats (Wistar TG)	6h–18h	24h	Pineal Gland	Higher at about 1/3^rd^ of DP, followed by a sharp decrease until the end of DP. Levels return to baseline level and stay stable during LP
Liu et al (2006) D [73]	Rats (PVG)	6h–18h	24h	Pineal Gland	Higher 1h after DP onset, followed by a decrease until the end of DP. Levels return to baseline level and stay stable during LP
Liu et al (2006) E [73]	Rats (LEW)	6h–18h	24h	Pineal Gland	Higher 3h–4h after DP onset, followed by decrease until the end of DP. Levels return to baseline level and stay stable during LP
Liu et al (2006) F [73]	Hamsters	6h–18h	24h	Pineal Gland	Peak at DP onset followed by an increase and fluctuations (less marked than in rats)
Garabette et al (2000) [74]	Rats	7h–19h	24h	Adjacent to SCN	Lower during DP. Higher during LP
Grossman et al (2000) A [75]	Hamsters	5h–19h	11h	Lateral Margin of SCN	Higher after DP onset. Stable during LP
Dudley et al (1998) A [76]	Hamsters	7h–22h	24h	Lateral Margin of the SCN	Peak at DP onset followed by gradual decrease. Stay stable during LP
Dudley et al (1998) B [76]	Hamsters	7h–22h	48h	Lateral Margin of the SCN	Peak 2h after DP onset, followed by gradual decrease. Stay stable during LP
Barassin et al (2002) [77]	Rats	12:12	17h	SCN or in Between SCN Nuclei	Peak at DP onset followed by decrease. Lower during LP
Knoch et al (2004) [78]	Hamsters	12:12	24h	SCN	Peak 2h after DP onset, followed by decrease. Lower during LP
Oshima et al (2003) [79]	Mice	6h–18h	24h	Hippocampus	Higher during DP, peak at onset and mid DP. Decrease during LP (but one peak mid LP)
Lopez-Rodriguez et al (2003) a [80]	Rats	1h–13h	24h	Posterior Hippocampus	Small peak at LP onset, yet fairly stable
Linthorst et al (1994) [81]	Rats	7h30–19h30	11h	Hippocampus	Peak at DP onset. Fairly stable during LP
Yang et al (2013) A [49]*	Mice (SERT +/+)	4h–16h	20h	Ventral Hippocampus and Ventral Striatum	Peak 3h after DP onset followed by a decrease. LP and rest of DP stable
Yang et al (2013) B [49]*	Mice (SERT +/–)	4h–16h	20h	Ventral Hippocampus and Ventral Striatum	Smaller peak 3h after DP onset followed by a sudden sharp decrease. LP and rest of DP stable. Or peak at 3h + peak 3h before LP onset. Or overall fluctuation
Yang et al (2013) C [49]*	Mice (SERT –/–)	4h–16h	20h	Ventral Hippocampus and Ventral Striatum	Gradual decrease during both DP and LP
Kalen et al (1989) [82]	Rats	12:12	24h	Caudal Hippocampus	Higher during DP, lower during LP
Penalva et al (2002) A [83]	Mice (CHR-R1 +/+)	6h–18h	18h	Dorsal Hippocampus	Higher during DP, lower during LP
Penalva et al (2002) B [83]	Mice (CHR-R1 +/–)	6h–18h	18h	Dorsal Hippocampus	Higher during DP, lower during LP
Penalva et al (2002) C [83]	Mice (CHR-R1 –/–)	6h–18h	18h	Dorsal Hippocampus	Higher during DP, lower during LP
Takahashi et al (1998) [84]	Rats	7h–17h	24h	Striatum	Higher during DP, stable during LP
Verhagen et al (2009) [64]	Rats	2h–14h	36h	Lateral to Nucleus Accumbens Shell	Higher during DP, lower during LP. Start increase 1h before DP onset, and reach its highest 5h after DP onset. Then decrease and reach nadir during mid-LP.
Izumo et al (2012) [66]	Rats	7h–19h	15h	Central Nucleus of the Amygdala	Peak at DP onset and mid DP followed each time by gradual decrease. Stable during LP
Smriga et al (2002) [85]	Rats	7h–19h	25h	Central Nucleus of the Amygdala	Peak at DP onset followed by gradual decrease. 1h before LP, increase to baseline level. Stable during LP
Dugovic et al (2009) [54]	Rats	6h–18h	6h	Prefrontal Cortex	Higher during DP, stable during LP
Barbier et al (2007) [55]	Rats	6h–18h	20h	Prefrontal Cortex	Stable
Jitsuki et al (2009) A [50]	Rats, male	5h–19h	24h	Medial Prefrontal Cortex	Fairly stable
Jitsuki et al (2009) B [50]	Rats, diestrous	5h–19h	24h	Medial Prefrontal Cortex	Higher during DP, lower during LP
Jitsuki et al (2009) C [50]	Rats, proestrous	5h–19h	24h	Medial Prefrontal Cortex	Higher during DP, lower during LP
Grossman et al (2004) [86]	Hamsters	14:10	24h	Margin of Thalamic Intergeniculate Leaflet	Higher 1h after DP onset, higher during DP Lower during LP, nadir mid LP
Sayer et al (1999) [87]	Rats	6h–18h	?	Anterior Hypothalamus	Stable during DP, slightly higher during LP
Fetissov et al (2000) 1 [65]	Rats	6h–18h	24h	Lateral hypothalamus	Stable apart from one peak during LP
Fetissov et al (2000) 2 [65]	Rats	6h–18h	24h	Ventromedial Hypothalamus	Peak 1h–2h after DP onset, followed by a return to baseline. Stable during rest of DP and LP

Each row represents one study (i.e. an experimental group within a publication) and a qualitative description of the results. Fluctuations are described as “higher” and “lower” disregarding actual magnitudes of changes. Rows are sorted by brain region. Lower case letters indicate separate publications from the same authors in the same year; upper cases letters represent separate groups within publications; numbers represent separate brain regions within animals. * These studies provided average monoamine concentrations pooled for several brain regions. Abbreviations: L/D cycle: Light-Dark Cycle; LP: Light Phase; DP: Dark Phase; LEW: Lewis; SD: Sprague-Dawley; TG: Transgenic; SERT: Serotonin Transporter; SCN: Suprachiasmatic Nucleus.

**Table 4:** Circadian rhythms in 5–HIAA levels.

5-HIAA – Circadian Rhythms					
Reference_ID	Animals	L/D Cycle	Duration	Brain Region	5–HIAA Levels

Glass et al (1993) a [88]	Hamsters	8h–22h	24h	Lateral margin of SCN	Peak at DP onset followed by a gradual decrease. Stable during LP, lower than DP
Luo et al (1999) A [89]	Hamsters, glucose intolerant	8h30–22h30 9h–23h	24h	Top of SCN	Increase during DP (peak 4h after DP onset), decreases during LP
Luo et al (1999) B [89]	Hamsters, glucose tolerant	8h30–22h30 9h–23h	24h	Top of SCN	Small peak during DP. Rather stable during LP
Barassin et al (2002) [77]	Rats	12:12	17h	SCN or in Between SCN Nuclei	Peak 4–6h after DP onset followed by decrease
Glass et al (1993) b [90]	Hamsters	7h–21h	24h	SCN	Peak 2h after DP, increase during DP, decrease during LP
Glass et al (1992) A [91]	Hamsters	8h–0h	24h	SCN	Peak 2h after DP onset, return to baseline at LP onset. Stable during LP except a decrease at 19h
Glass et al (1992) B [91]	Hamsters	8h–0h	24h	Lateral Margin of the SCN	Peak 2h after DP onset, return to baseline at LP onset. Stable during LP except a decrease at 19h
Luo et al (2000) [92]	Hamsters	0h–14h	24h	SCN	Peak at DP onset and nadir 4h DP onset. Stable during the rest of sampling time.
Glass et al (1993) c [45]	Hamsters	8h–0h	24h	SCN	Peak at DP onset, increases during DP, decreases during LP
Castaneda et al (2004) A1 [59]	Rats	20h–8h	30h	Striatum	Slight increase during DP, slight decrease during LP
Castaneda et al (2004) B1 [59]	Rats	6h DP–24h LP	30h	Striatum	Lower during DP, higher during LP
Sano et al (1992) A [47]	Rats, young animals	6h–18h	24h	Striatum	Nadir at DP onset, then increase gradually until the end of DP. Start of LP decrease gradually.
Sano et al (1992) B [47]	Rats, old animals	6h–18h	24h	Striatum	Stable
Sano et al (1992) C [47]	Rats, enriched media	6h–18h	24h	Striatum	Stable
Sano et al (1992) D [47]	Rats, isolated	6h–18h	24h	Striatum	Stable
Smith et al (1992) [46]	Rats	7h–19h	18h	Striatum	Stable
Nakayama et al (2002) [93]	Rats	8h–20h	24h	Striatum	Higher during DP, lower during LP
Takahashi et al (1998) [84]	Rats	7h–19h	24h	Striatum	Higher during DP, lower during LP
Castaneda et al (2004) A2 [59]	Rats	20h–8h	30h	Nucleus Accumbens	Higher during DP, lower during LP
Castaneda et al (2004) B2 [59]	Rats	6h DP–24h LP	30h	Nucleus Accumbens	Inconsistent during DP (fluctuation up and down), lower during LP
Paulson et al (1994) 2 [61]	Rats	6h–20h	20h	Nucleus Accumbens	Higher during DP, lower during LP
Paulson et al (1996) [62]	Rats	6h–20h	18h20	Nucleus Accumbens	Slightly higher during DP, lower during LP
Verhagen et al (2009) [64]	Rats	2h–14h	36h	Lateral to Nucleus Accumbens Shell	Higher during DP (peak around the end of DP), lower during LP
Paulson et al (1994) 1 [61]	Rats	6h–20h	20h	Caudate Nucleus	Higher during DP, lower during LP
Paulson et al (1996) [62]	Rats	6h–20h	18h20	Dorsolateral Caudate Nucleus	Higher during DP, lower during LP
Oshima et al (2003) [79]	Mice	6h–18h	24h	Hippocampus	Higher during DP, lower during LP
Nakayama et al (2002) [93]	Rats	8h–20h	24h	Hippocampus	Stable
Linthorst et al (1994) [81]	Rats	7h30–19h30	11h	Hippocampus	Fairly stable, higher at DP onset (slightly)
Kalen et al (1989) [82]	Rats	12:12	24h	Caudal Hippocampus	Stable, apart from a peak at the end of DP
Penalva et al (2002) A [83]	Mice, (CHR–R1 +/+)	6h–18h	18h	Dorsal Hippocampus	Higher during DP, lower during LP
Penalva et al (2002) B [83]	Mice, (CHR–R1 +/–)	6h–18h	18h	Dorsal Hippocampus	Higher during DP, lower during LP
Penalva et al (2002) C [83]	Mice, (CHR–R1 –/–)	6h–18h	18h	Dorsal Hippocampus	Higher during DP, lower during LP (higher levels than other mice)
Glass et al (1992) C [91]	Hamsters	8h–0h	24h	Preoptic Area	Peak at DP onset followed by gradual decrease. Stable during LP
Ezrokhi et al (2014) A [94]	Rats (CTL)	5h–19h	24h	Ventromedial Hypothalamus	Gradual decrease (start at LP)
Ezrokhi et al (2014) B [94]	Rats (SHR), treated with vehicle	5h–19h	24h	Ventromedial Hypothalamus	Higher during DP, lower during LP
Luo et al (1998) A [95]	Hamsters, glucose tolerant	8h30–22h30	25h	Ventromedial Hypothalamus	Lower level than intolerant group. Higher during DP with a peak at the end. Lower during LP
Luo et al (1998) B [95]	Hamsters, glucose intolerant	8h30–22h30	25h	Ventromedial Hypothalamus	Higher level and more fluctuations than tolerant group. Levels increases during DP with a peak at the end. Lower levels during LP
Luo et al (1998) C [95]	Hamsters (CTL)	8h30–22h30	25h	Ventromedial Hypothalamus	Fairly stable, higher during DP (slightly)
Stanley et al (1989) a [96]	Rats	9h–21h	24h	Paraventricular Nucleus	Peak 1h after DP onset followed by sudden decrease. Lower during LP
Glass et al (1992) D [91]	Hamsters	8h–0h	24h	Posterior Hypothalamus	Peak at DP onset followed by gradual decrease Stable during LP
Gonzales-Pina et al (2003) [97]	Rats	12:12	24h	Dorsal Raphe	Higher during DP, lower during LP
Azekawa et al (1991) [71]	Rats	7h–19h	24h	Pineal gland	Peak at DP onset followed by strong decrease and then a gradual increase until the end of DP. Lower levels during LP
Nakayama et al (1993) [56]	Rats	8h–20h	24h	Medial Prefrontal Cortex	Higher during DP, lower during LP
Nakayama et al (2002) [93]	Rats	8h–20h	24h	Medial Prefrontal Cortex	Higher during DP, lower during LP

Each row represents one study (i.e. an experimental group within a publication) and a qualitative description of the results. Fluctuations are described as “higher” and “lower” disregarding actual magnitudes of changes. Rows are sorted by brain region. Lower case letters indicate separate publications from the same authors in the same year; upper cases letters represent separate groups within publications; numbers represent separate brain regions within animals. Abbreviations: 5-HIAA: 5-hydroxyindoleacetic acid; L/D Cycle: Light-Dark Cycle; LP: Light Phase; DP: Dark Phase; SCN: Suprachiasmatic Nucleus; CTL: Control, SHR: Spontaneously Hypertensive Rats; CHR–R1: Corticotropin-Releasing Hormone Receptor 1.

**Table 5:** Circadian rhythms in noradrenaline levels.

Noradrenaline-Circadian Rhythms					

Reference_ID	Animals	L/D Cycle	Duration	Brain Region	Noradrenaline Levels

Barbier et al (2007) [55]	Rats	6h–18h	20h	Prefrontal Cortex	Higher during DP, stable during LP
Dugovic et al (2009) [54]	Rats	6h–18h	6h	Prefrontal Cortex	Higher during DP, stable during LP
Robinson et al (1991) [57]	Sheep	natural	20h	Preoptic Area	Decrease gradually
Alfinito et al (2009) [58]	Rats	12:12	12h30	Preoptic Area	Higher during DP, stable during LP
Mitome et al (1994) a [37]	Rats	6h–18h	52h	Paraventricular Nucleus	Higher during DP, lower during LP
Stanley et al (1989) b [98]	Rats	9h–21h	48h	Paraventricular Nucleus	Peak 1h after DP onset, followed by sudden decrease, until a second smaller peak at 3h before LP. Lower levels during LP
Mitome et al (1994) b [99]	Rats	6h–18h	54h	Paraventricular Nucleus	Higher during DP, lower during LP
Morien et al (1995) A [100]*	Rats	7h–19h	24h	Paraventricular Nucleus	Peak 1h and 8h after DP onset. Higher during DP, lower during LP
Smriga et al (2000) b [101]	Rats	7h–19h	24h	Lateral Hypothalamus	Gradual increase from baseline during LP Peak at DP onset followed by sudden decrease and return to baseline
Smriga et al (2000) a [102]	Rats	7h–19h	26h	Ventral Hypothalamus	Higher during DP, lower during LP
Kalen et al (1989) [82]	Rats	12:12	24h	Caudal Hippocampus	Higher during DP, lower during LP
Drijfhout et al (1996) [103]	Rats	6h–18h	16h	Pineal gland	Peak 1–3h after DP onset, decrease 2h before LP Higher during DP, lower during LP
Morien et al (1995) B [100]*	Rats	7h–19h	24h	Septal Nuclei and the Ventromedial Thalamus	Stable

Each row represents one study (i.e. an experimental group within a publication) and a qualitative description of the results. Fluctuations are described as “higher” and “lower” disregarding actual magnitudes of changes. Rows are sorted by brain region. Lower case letters indicate separate publications from the same authors in the same year; upper cases letters represent separate groups within publications; numbers represent separate brain regions within animals. *These studies provided average monoamine concentrations pooled for several brain regions. Abbreviations: L/D Cycle: Light-Dark Cycle; LP: Light Phase; DP: Dark phase.

**Table 6:** Circadian rhythms in adrenaline levels.

Adrenaline-Circadian Rhythms					

Reference_ID	Animals	L/D Cycle	Duration	Brain Region	Adrenaline Levels

Robinson et al (1991) [57]	Sheep	Natural cycle	20h	Preoptic Area	Stable

Abbreviations: L/D Cycle: Light/Dark Cycle; DP: Dark-Phase; LP: Light-Phase.

**Table 7:** Dopamine levels during naturally occurring sleep stages.

Dopamine-Sleep				

Reference	Animals	L/D Cycle	Brain Region	Dopamine Levels

Orosco et al (1995) [104]	Rats	6h–18h	PVN/VMN	2 days measurement with different observation Day1: W and REM high, SWS lower/ Day2: W and REM low with SWS high
Nicolaidis et al (2001) A [105]	Rats	?	PVN/VMN	Levels increases from SWS to REM and from REM to W. Levels decreases from W to SWS
Shouse et al (2000) a 1 [106]	Cats	?	Amygdala	Stable during all stages (AW, QW, SWS and REM)
Shouse et al (2001) a 1 [107]	Cats	?	Amygdala	Stable during wake and sleep
Shouse et al (2001) b 1 [108]	Cats	?	Amygdala	Stable during wake and sleep
Shouse et al (2000) a 2 [106]	Cats	?	Locus Coeruleus	Stable during all stages (AW, QW, SWS and REM)
Shouse et al (2001) a 1 [107]	Cats	?	Locus Coeruleus	Stable during wake and sleep
Shouse et al (2001) b 2 [108]	Cats	?	Locus Coeruleus	Stable during wake and sleep
Lena et al (2005) 1 [109]	Rats	8h–20h	Medial Prefrontal Cortex	W: high level, SWS: low level, REM: in between
De Saint Hilaire (2000) [110]	Rats	6h–18h	Prefrontal Cortex	W: lower, SWS: high. Higher in REM when followed by W
Nicolaidis et al (2001) B [105]	Rats	?	Prefrontal Cortex	W to SWS: decrease, SWS to W: increases
Lena et al (2005) 2 [109]	Rats	8h–20h	Nucleus Accumbens	W and REM: high, SWS: low

Each row represents one study (i.e. an experimental group within a publication) and a qualitative description of the results. Fluctuations are described as “higher” and “lower” disregarding actual magnitudes of changes. Rows are sorted by brain region. Lower case letters indicate separate publications from the same authors in the same year; upper cases letters represent separate groups within publications; numbers represent separate brain regions within animals. Abbreviations: W: Wake; SWS: Slow Wave Sleep; REM: Rapid Eye Movements Sleep; PVN: Paraventricular Nucleus; VMN: Ventromedial Hypothalamic Nucleus.

**Table 8:** DOPAC levels during naturally occurring sleep stages.

DOPAC-Sleep				

Reference	Animals	L/D Cycle	Brain Region	DOPAC Levels

De Saint Hilaire (2000) [110]	Rats	6h–18h	Prefrontal Cortex	Stable
Orosco et al (1995) [104]	Rats	6h–18h	PVN/VMN	W: low, SWS: intermediate, REM: high
Nicolaidis et al (2001) A [105]	Rats	?	PVN/VMN	From SWS to W: decrease, from SWS to REM: increase, from REM to W: increase

Each row represents one study (i.e. an experimental group within a publication) and a qualitative description of the results. Fluctuations are described as “higher” and “lower” disregarding actual magnitudes of changes. Rows are sorted by brain region. Lower case letters indicate separate publications from the same authors in the same year; upper cases letters represent separate groups within publications; numbers represent separate brain regions within animals. Abbreviations: DOPAC: 3,4–Dihydroxyphenylacetic acid; W: Wake; SWS: Slow Wave Sleep; REM: Rapid Eye Movements Sleep; PVN: Paraventricular Nucleus; VMN: Ventromedial Hypothalamic Nucleus.

**Table 9:** Serotonin levels during naturally occurring sleep stages.

Serotonin-Sleep				

Reference	Animals	L/D Cycle	Brain Region	Serotonin Levels

Orosco et al (1995) [104]	Rats	6h–18h	PVN/VMN	W: high, SWS: intermediate, REM: low
Nicolaidis et al (2001) A [105]	Rats	?	PVN/VMN	W: high, SWS: low
Wilkinson et al (1991) [111]	Cats	?	Preoptic Area/Anterior Hypothalamus	W: high, SWS: low
Python et al (2001) [112]	Rats	8h–20h	Preoptic Area	W: high, SWS: intermediate, REM: low. SWS after REM showed no strong fluctuation, but when W after REM levels showed a strong increase
Shouse et al (2000) a 1 [106]	Cats	?	Amygdala	W: high, SWS: intermediate, REM: low
Shouse et al (2001)a 1 [107]	Cats	?	Amygdala	W: high, SWS: low
Shouse et al (2001) b 1 [108]	Cats	?	Amygdala	W: high, SWS: intermediate, REM: low
Shouse et al (2000) a 2 [106]	Cats	?	Locus Coeruleus	W: high, SWS: intermediate, REM: low
Shouse et al (2001) a 2 [107]	Cats	?	Locus Coeruleus	W: high, SWS: low
Shouse et al (2001) b 1 [108]	Cats	?	Locus Coeruleus	W: high, SWS: intermediate, REM: low
Park et al (1999) [113]	Rats	7h–19h	Posterior Hippocampus	W: high, SWS and REM: low
Gronli et al (2007) [114]	Rats	7h–19h	Hippocampus	W and SWS: high, REM: low
Bjorvatn et al (2002) A1 [115]	Rats	6h–18h	Ventral Hippocampus	W: high, Sleep: low
Penalva et al (2003) A [116]	Rats	7h30–19h30	Dorsal Hippocampus	W: high, SWS: low, REM: low
Fiske et al (2006) 1 [117]	Rats	6h–18h	Dorsal Raphe	W: high, SWS and REM: low
Fiske et al (2008) 1 [118]	Rats	6h–18h	Dorsal Raphe	W: high, SWS and REM: low
Portas et al (1994) [119]	Cats	Constant light	Dorsal Raphe	W: high, SWS: intermediate, REM: low
Portas et al (1996) [120]	Cats	Constant light	Dorsal Raphe	W: high, SWS: intermediate, REM: low
Portas et al (1998) 1 [121]	Rats	6h–18h	Dorsal Raphe	W: high, SWS: intermediate, REM: low
De Saint Hilaire et al (2000) [110]	Rats	6h–18h	Prefrontal Cortex	W: high, SWS: intermediate, REM: low. Except, 5–HT increases in REM if followed by W
Nicolaidis et al (2001) B [105]	Rats	?	Prefrontal Cortex	W increases before a SWS stage. From SWS to W decrease after a long SWS period.
Portas et al (1998) 2 [121]	Rats	6h–18h	Frontal cortex	W: high, SWS: intermediate, REM: low
Mukaida et al (2007) [122] Ɨ	Rats	7h–19h	Frontal cortex	W: high, SWS: lower
Fiske et al (2008) 2 [118]	Rats	6h–18h	Frontal cortex	Stable
Bjorvatn et al (2002) A2 [115]	Rats	6h–18h	Frontal cortex	W: high, Sleep: low
Zeitzer et al (2002) [41]	Human	L/D cycle of the season	Lateral Ventricle	W: high, SWS: intermediate, REM: low From stage 2 to REM: decrease/from REM to stage 2: increase
McCarley et al (2004) [123]	?	?	PPT	W: high, SWS: intermediate, REM: low
Strecker et al (1999) [124]	Cats	?	PPT	W: high, SWS: intermediate, REM: low
Fiske et al (2006) 2 [117]	Rats	6h–18h	Frontal cortex	W: high, SWS: low, REM: intermediate
Lapierre et al (2012) [125]	Seals	?	Cortex	W: high, SWS: intermediate, REM: low
Lapierre et al (2013) a [126]	Seals	8h–20h	Cerebral cortex	W: high, BSWS: the lowest, REM: low
Lyamin et al (2016) A [127]*	Seals	8h–20h	Occipital cortex and Frontal cortex	W: high, SWS: intermediate, REM: low. Same decrease was seen in seals specific sleep stages (USWS (right and left), BSWS)
Blanco-Centurion et al (2001) A [128]	Rats	8h–20h	Gigantocellular reticular nucleus	W: high, SWS: intermediate, REM: low
Iwakiri et al (1993) [129]	Cats	?	Medial Pontine Reticular Formation	W: high, SWS: intermediate, REM: low
Lyamin et al (2016) C [127]	Seals	8h–20h	Thalamus	W: high, SWS: intermediate, REM: low
Lyamin et al (2016) D [127]	Seals	8h–20h	Caudate nucleus	W: high, SWS: intermediate, REM: low

Each row represents one study (i.e. an experimental group within a publication) and a qualitative description of the results. Fluctuations are described as “higher” and “lower” disregarding actual magnitudes of changes. Rows are sorted by brain region. Lower case letters indicate separate publications from the same authors in the same year; upper cases letters represent separate groups within publications; numbers represent separate brain regions within animals. Ɨ Anaesthesia was applied during baseline: 6 L/min mixture of 25% oxygen and75% nitrogen. All the other studies measured natural sleep. *These studies provided average monoamine concentrations pooled for several brain regions. Abbreviations: W: Wake; SWS: Slow Wave Sleep; REM: Rapid Eye Movements Sleep; PVN: Paraventricular Nucleus; VMN: Ventromedial Hypothalamic Nucleus; PPT: Pedunculopontine Tegmental Nucleus.

**Table 10:** 5-HIAA levels during naturally occurring sleep stages.

5-HIAA-Sleep				

Reference	Animals	L/D Cycle	Brain Region	5-HIAA Levels

De Saint Hilaire (2000) [110]	Rats	6h–18h	Prefrontal Cortex	Stable
Orosco et al (1995) [104]	Rats	6h–18h	PVN/VMN	1^st^ day: W: intermediate, SWS: low, REM: high 2^nd^ day: W: high, SWS: intermediate, REM: low
Nicolaidis et al (2001) A [105]	Rats	?	PVN/VMN	W: High, Sleep: Lower. W is lower if preceded by REM.
Portas et al (1994) [119]	Cats	Constant light	Dorsal Raphe	Stable
Iwakiri et al (1993) [129]	Cats	?	Medial Pontine Reticular Formation	Stable

Each row represents one study (i.e. an experimental group within a publication) and a qualitative description of the results. Fluctuations are described as “higher” and “lower” disregarding actual magnitudes of changes. Rows are sorted by brain region. Lower case letters indicate separate publications from the same authors in the same year; upper cases letters represent separate groups within publications; numbers represent separate brain regions within animals. Abbreviations: 5-HIAA: 5-hydroxyindoleacetic acid; W: Wake; SWS: Slow Wave Sleep; REM: Rapid Eye Movements Sleep; PVN: Paraventricular Nucleus; VMN: Ventromedial Hypothalamic Nucleus.

**Table 11:** Noradrenaline levels during naturally occurring sleep stages.

Noradrenaline-Sleep				

Reference	Animals	L/D Cycle	Brain Region	Noradrenaline Levels

Orosco et al (1995) [104]	Rats	6h–18h	PVN/VMN	1^st^ day: W: high, SWS: low, REM: intermediate 2^nd^ day W: high, SWS: intermediate, REM: low
Nicolaidis et al (2001) A [105]	Rats	?	PVN/VMN	W: high, SWS: intermediate, REM: low. If SWS followed by W or REM: increase, while if REM or W is followed by SWS: decrease
Lyamin et al (2016) B [127]	Seals	8h–20h	Hypothalamus	W: high, SWS: intermediate, REM: low
Shouse et al (2000) a 1 [106]	Cats	?	Amygdala	W: high, SWS: intermediate, REM: low
Shouse et al (2000) b 1 [130]	Cats	?	Amygdala	W: high, SWS: low
Shouse et al (2001) a 1 [107]	Cats	?	Amygdala	W: high, SWS: low
Shouse et al (2001) b 1 [108]	Cats	?	Amygdala	W: high, SWS: intermediate, REM: low
Park et al (2002) [131]	Rats	7h–19h	Amygdala	W: high, SWS: low, REM: lower
Shouse et al (2000) a 2 [106]	Cats	?	Locus Coeruleus	W: high, SWS: intermediate, REM: low
Shouse et al (2000) b 2 [130]	Cats	?	Locus Coeruleus	W: high, Sleep: low
Shouse et al (2001) a 2 [107]	Cats	?	Locus Coeruleus	W: high, Sleep: low
Shouse et al (2001) b 2 [108]	Cats	?	Locus Coeruleus	W: high, SWS: intermediate, REM: low
Bellesi et al (2016) A1	Mice	8h–20h	Medial Prefrontal Cortex	W: high, Sleep: low
Lena et al (2005) 1 [109]	Rats	8h–20h	Medial Prefrontal Cortex	W: high, SWS: intermediate, REM: low
De Saint Hilaire et al (2000) [110]	Rats	6h–18h	Prefrontal Cortex	W: lower, SWS: high (relatively stable)
Lena et al (2005) 2 [109]	Rats	8h–20h	Nucleus Accumbens	W: high, SWS: intermediate, REM: low
Lapierre et al (2013) b [132]	Seals	?	Cortex	W: high, SWS: intermediate, REM: low.
Lyamin et al (2016) A [127]*	Seals	8h–20h	Occipital cortex, frontal cortex	W: high, SWS: intermediate, REM: low. Same decrease was seen in seals specific sleep stages (USWS (right and left), BSWS)
Bellesi et al (2016) A2	Mice	8h–20h	M1	W: high, Sleep: low

Each row represents one study (i.e. an experimental group within a publication) and a qualitative description of the results. Fluctuations are described as “higher” and “lower” disregarding actual magnitudes of changes. Rows are sorted by brain region. Lower case letters indicate separate publications from the same authors in the same year; upper cases letters represent separate groups within publications; numbers represent separate brain regions within animals. * These studies provided average monoamine concentrations pooled for several brain regions. Abbreviations: W: Wake; SWS: Slow Wave Sleep; REM: Rapid Eye Movements Sleep; PVN: Paraventricular Nucleus; VMN: Ventromedial Hypothalamic Nucleus; M1: Primary Motor Cortex.

**Table 12:** Dopamine and sleep deprivation.

Dopamine-SD					

Reference_ID	Animals	SD Methods	Duration	Brain Region	Dopamine levels during/after SD

Murillo-Rodriguez et al (2016) [134]	Rats	-Stroking fur with paint brush -Light noise in the cage -Tapping -Placing object in the cage	6h	Nucleus Accumbens	Increase after SD

Each row represents one study (i.e. an experimental group within a publication) and a qualitative description of the results. Abbreviation: SD: Sleep Deprivation.

**Table 13:** DOPAC and sleep deprivation.

DOPAC-SD					

Reference_ID	Animals	SD Methods	Duration	Brain Region	DOPAC levels during/after SD

Zant et al (2010) [135]	Rats	Gentle handling	6h	Basal Forebrain	Increase during SD, decrease to baseline levels during sleep recovery
Zant et al (2011) [136]	Rats	-Gentle handling including placing objects in the cage	6h	Basal Forebrain	Increase during 3 first hours of SD, then plateau. It decreases to baseline levels during sleep recovery

Each row represents one study (i.e. an experimental group within a publication) and a qualitative description of the results. Abbreviations: DOPAC: 3,4-Dihydroxyphenylacetic acid; SD: Sleep Deprivation.

**Table 14:** Serotonin and sleep deprivation.

Serotonin-SD					

Reference_ID	Animals	SD Methods	Duration	Brain Region	Serotonin levels during/after SD

Bjorvatn et al (2002) B1 [115]	Rats	-Gentle sensory stimulation (knocking on the plexiglas door, opening the door, gentle handling)	8h30	Ventral hippocampus	Decrease during SD
Lopez-Rodriguez et al (2003) a [80]	Rats	Modified disk-over-water	24h	Posterior Hippocampus	Increase during SD and remain high during recovery
Lopez-Rodriguez et al (2003) b [133]	Rats	Small platform (6cm) in tank filled with water (REM deprivation)	24h but measurement for 11h	Posterior Hippocampus	Increase during SD and decrease below baseline during recovery
Penalva et al (2003) B [116]	Rats	-Introducing or removing objects -Shaking the cage slightly	4h	Dorsal hippocampus	Increase during SD. During recovery time, levels are high during W and low during REM sleep.
Penalva et al (2003) C [116]	Rats	-Introducing or removing objects -Shaking the cage slightly	4h	Dorsal hippocampus	Increase during SD. During recovery time, levels are high during W and low during REM sleep.
Bjorvatn et al (2002) B2 [115]	Rats	-Gentle sensory stimulation (knocking on the plexiglas door, opening the door, handling)	8h30	Frontal Cortex	Decrease during SD
Blanco-Centurion et al (2001) B [128]	Rats	Platform (6.5cm) surrounded by water (REM deprivation)	92h	Gigantoreticular Cellular Nucleus	Decrease (factor 100) during SD and remain low during recovery
Grossman et al (2000) B [75]	Hamsters	-Continuous gentle handling -Light puffs of air	3h (red dim light)	Lateral Margin of SCN	Increase during SD, decreases during recovery but slight increase at the end.
Grossman et al (2000) C [75]	Hamsters	-Continuous handling-Light puffs of air	3h	Lateral Margin of SCN	Increase during SD, highest peak at the end of SD. Decreases to baseline levels during recovery
Murillo-Rodriguez et al (2016) [134]	Rats	-Stroking fur with paint brush-Light noise in the cage-Tapping-Placing object in the cage	6h	Nucleus Accumbens	Increase after SD

Each row represents one study (i.e. an experimental group within a publication) and a qualitative description of the results. Rows are sorted by brain region. Lower case letters indicate separate publications from the same authors in the same year; upper cases letters represent separate groups within publications; numbers represent separate brain regions within animals. Abbreviations: SD: Sleep Deprivation; SCN: Suprachiasmatic Nucleus.

**Table 15:** 5-HIAA and sleep deprivation.

5-HIAA-SD					

Reference_ID	Animals	SD Methods	Duration	Brain Region	5-HIAA levels during/after SD

Zant et al (2010) [135]	Rats	Gentle handling	6h	Basal Forebrain	Increase during SD and return to baseline level during recovery
Zant et al (2011) [136]	Rats	-Gentle handling -Placing object in the cage	6h	Basal Forebrain	Increase during SD and return to baseline level during recovery
Blanco-Centurion et al (2001) B [128]	Rats	Platform (6.5cm) surrounded by water (REM deprivation)	92h	Gigantoreticular Cellular Nucleus	Decrease during SD, and increase during recovery

Each row represents one study (i.e. an experimental group within a publication) and a qualitative description of the results. Rows are sorted by brain region. Lower case letters indicate separate publications from the same authors in the same year; upper cases letters represent separate groups within publications; numbers represent separate brain regions within animals. Abbreviations: SD: Sleep Deprivation; 5-HIAA: 5-Hydroxyindoleacetic acid.

**Table 16:** Noradrenaline and sleep deprivation.

Noradrenaline-SD					

Reference_ID	Animals	SD Methods	Duration	Brain Region	Noradrenaline levels during/after SD

Bellesi et al (2016) B1	Mice	-Exposure to novel objects	6h	Medial Prefrontal Cortex	Increase during SD and slightly decrease at the end
Bellesi et al (2016) B2	Mice	-Exposure to novel objects	6h	M1	Increase during SD
Murillo-Rodriguez et al (2016) [134]	Rats	-Stroking fur with paint brush -Light noise in the cage-Tapping-Placing object in the cage	6h	Nucleus Accumbens	Increase after SD

Each row represents one study (i.e. an experimental group within a publication) and a qualitative description of the results. Rows are sorted by brain region. Lower case letters indicate separate publications from the same authors in the same year; upper cases letters represent separate groups within publications; numbers represent separate brain regions within animals. Abbreviations: SD: Sleep Deprivation; M1: Primary Motor Cortex.

**Table 17:** Adrenaline and sleep deprivation.

Adrenaline-SD					

Reference_ID	Animals	SD Methods	Duration	Brain Region	Adrenaline levels during/after SD

Murillo-Rodriguez et al (2016) [134]	Rats	-Stroking fur with painting brush-Light noise during the cage-Tapping-Placing object in the cage	6h	Nucleus Accumbens	Increase after SD

Abbreviations: SD: Sleep Deprivation.

**Table 18:** Summary of medians, interquartile ranges and Friedman’s ANOVA test statistics for each compound analysed during the different phases of the experiment.

Analyte	Phase	Median (nmol/L)	IQR 25%	IQR 75%	Test statistics

5-HT	Light	1.56	1.17	6.88	χ^2^(3) = 5.694 p = 0.127
	Dark	0.92	0.61	2.21	
	SD	0.96	0.37	1.67	
	Recovery	1.19	0.44	4.60	

5-HIAA	Light	86.67	61.21	60.71	χ^2^(3) = 6.60 p = 0.086
	Dark	42.56	20.80	98.86	
	SD	103.32	72.68	55.56	
	Recovery	104.07	86.46	33.14	

5-HTP	Light	2.17	0.35	2.21	χ^2^(3) = 4.92 p = 0.178
	Dark	1.57	0.24	1.45	
	SD	2.43	0.46	0.95	
	Recovery	1.20	0.89	1.23	

DA	Light	0.49	0.19	0.15	χ^2^(3) = 5.40 p = 0.145
	Dark	0.26	0.12	0.12	
	SD	0.34	0.10	0.22	
	Recovery	0.30	0.15	0.12	

DOPAC	Light	2.10	0.97	2.47	χ^2^(3) = 8.846 p = 0.037
	Dark	1.70	0.67	3.35	
	SD	2.06	0.84	4.70	
	Recovery	1.33	0.36	1.36	

NA	Light	0.28	0.14	1.00	χ^2^(3) = 7.145 p = 0.067
	Dark	0.47	0.300	0.38	
	SD	0.31	0.19	0.44	
	Recovery	0.11	0.01	0.09	

ADRE	Light	0.18	0.06	0.04	χ^2^(3) = 1.8 p = 0.615
	Dark	0.21	0.10	0.06	
	SD	0.28	0.14	0.15	
	Recovery	0.23	0.11	0.09	

Friedman’s ANOVA’s were performed to compare concentrations (nmol/L) between the different phases. For 5-HT, light phase n = 7, dark phase and SD n = 8, and recovery n = 9. For 5-HIAA, light phase, dark phase, and SD n = 11, recovery n = 10. For 5-HTP, light phase n = 9, dark phase, SD, and recovery n = 8. For dopamine, all phases n = 11. For DOPAC, light phase and recovery n = 9, dark phase n = 7, SD n = 8. For noradrenaline, all phase n = 11. For adrenaline, light phase, SD and recovery n = 10, dark phase n = 9. Numbers of observations vary because of missing samples (temporarily obstructed flow) and some concentrations being below HPLC detection limits. Abbreviations: 5-HT: Serotonin; 5-HIAA: 5-Hydroxyindoleacetic Acid; 5-HTP: 5-Hydroxytryptophan; DA: Dopamine; DOPAC: 3,4-Dihydroxyphenylacetic acid; NA: Noradrenaline; ADRE: Adrenaline; IQR: Inter Quartile Range.

**Figure 4:**
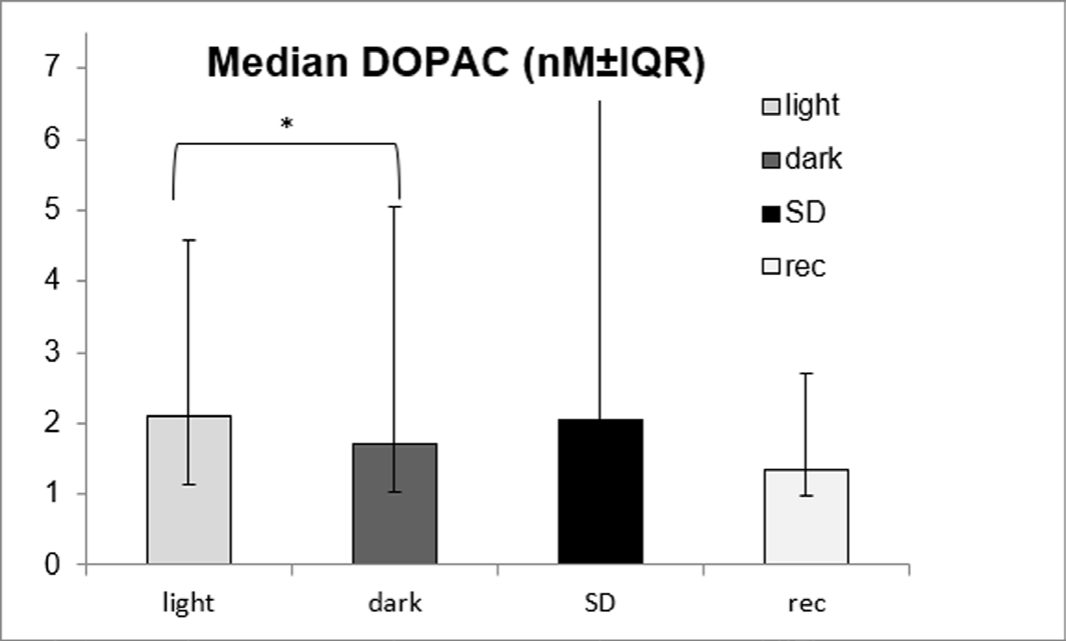
Median DOPAC dialysates concentrations in (nM) ± inter quartile range. Light: 12h of baseline during the light phase; dark: 12h of baseline during the dark phase; SD: 12h of sleep deprivation during the light phase and rec: recovery for 12h. *Wilcoxon signed rank test: T = 0, p = 0.018.

For the experimental data on monoamine levels in mPFC, we tabulated concentrations (nmol/L) and statistics for all compounds (Table 18) and present the findings for DOPAC in a figure (Figure 4).

### Systematic review

#### Search and selection

Our search retrieved 2662 publications; 1195 from Medline and 1467 from EMBASE. After duplicate removal, 1550 publications remained for title abstract screening, and thereafter, 1170 for full text screening. From these, 94 were included. Screening of the reference lists only resulted in one additional publication [45]. The flow of included and excluded publications is presented in Figure 1.

Data were extracted from the 94 included publications, which could comprise multiple “studies” as detailed in the methods.

#### Study characteristics and quality assessment

The 94 included publications comprised 89 full papers, 4 conference abstracts, and 1 review containing otherwise unpublished data. Of the 89 full texts, 11 described 2 different experiments, 18 more than one experimental group and 16 simultaneous measurements within one animal. The 152 resulting studies describe CR experiments [93], sleep experiments [45] or SD experiments [14].

#### Animals

Species was reported for 151 studies (99.34%). 95 Studies (62.5%) were on rats; 54 on Wistars, 31 on Sprague-Dawleys, 2 on Lewis, 2 on Holtzman, 2 on lean Zucker, 1 on PVG and 1 on Spontaneously Hypertensive rats. Hamsters were used in 21 studies (13.72%); 15 used Syrians, 4 Siberians, and 1 Djungarian. Cats and mice were both used in 13 studies (8.5% each). The strain of mice was C57BL/6 (5) and mutant (7; for SERT, CHR-R1 and DAT genes). The remaining studies were on seals (7), sheep (1), and one human (1). The sex of the animals was reported in 135 studies (88.82%); most reported using males only (113), only few used females only (10) or both genders (12). Forty-one studies (26.8%) reported both the animals’ age and weight; 101 (66.45%) reported one of the two [64 studies only weight, 37 studies only age). Groups sizes were reported for 139 studies (91.45%) and varied from 1–133 animals.

#### Experimental set-up and microdialysis

Length of light and dark phase (L/D cycle) was reported for 133 studies (87.5%), actual clock times were specified for 129. Overall, 102 studies used a 12:12 LD cycle, 20 a 14:10 LD cycle, 5 a 16:10 LD cycle and 2 microdialyzed over 6h of dark and 24h of light.

Post-surgical recovery time was reported for 124 studies (81.58%) and ranged from 24h to 3 weeks. Probe length was reported for 135 studies (88.81%), and probe/membrane type for 134 (88.16%). Perfusion matrix (e.g. aCSF, Ringer) and perfusion rate (0,12–3 μL/min excluding one study [46] using slow perfusion [57 nl/min]) were reported in 149 studies (98.03%). Nine studies (7 CR studies and 2 SD studies) used reuptake inhibitors in the perfusion matrix; all of them for serotonin. Sample bin time was reported for 148 studies (97.37%) and fluctuated from 3 min to 2h. Probe recovery (2.4%–72 ± 3%) was reported for 55 studies (36.2%); 53 provided actual values, 2 reported methods to determine recovery without values. Histological verification of probe placement was described for 110 studies (72.37%) and one study verified probe placement by CT-scan [41].

Sample analysis was reported for 148 studies (97.37%); 146 using High Performance Liquid Chromatography (HPLC) and 2 using capillary electrophoresis. Monoamines were measured k = 86 studies for serotonin, k = 52 studies for 5-HIAA, k = 41 studies for dopamine, k = 35 studies for noradrenaline, k = 25 studies DOPAC, k = 2 studies for adrenaline. 5-HTP was not measured in our sample of studies.

#### General reporting quality

Approval by an ethical committee was reported for 80 studies (52.63%). Randomization of at least one study stage was reported for 13 studies (8.55%) and power analysis for only 4 studies (2.63%). Authors from 26 studies (17.11%) declared not having any conflicts of interests, authors from 2 clearly stated a conflict of interest. Funding source was mentioned for 98 studies (64.47%).

#### Monoamine measurements and circadian rhythms

CRs in monoamine concentrations as described in the included studies are described by monoamine in Tables 1, 2, 3, 4, 5, 6. Monoamine levels fluctuate over the dark and light phases. Patterns depend on the brain area and monoamine studied. We describe the findings with 3 general patterns: pattern 1: monoamine levels are higher during the dark phase and lower during the light phase; pattern 2: levels peak at or around dark phase onset; and pattern 3: levels remain stable during dark and light. Dopamine, DOPAC and noradrenaline mostly followed pattern 1. Serotonin and 5-HIAA mostly followed pattern 2 and occasionally pattern 1. Dopamine, noradrenaline and adrenaline sporadically followed pattern 3.

The patterns may differ by brain region; for some brain regions, a specific pattern was observed, while for others, patterns varied. For instance, pattern 1 was observed for dopamine and DOPAC levels in the caudate putamen, for DOPAC and 5-HIAA levels in the nucleus accumbens, and for serotonin, 5-HIAA, and noradrenaline levels in the hippocampus. In the striatum pattern 1 and 3 have been observed for dopamine and 5-HIAA; for DOPAC only pattern 1 has been described.

Likewise, pattern 2 was observed for serotonin levels in the amygdala and pineal gland, and for serotonin and 5-HIAA levels in the suprachiasmatic nucleus. In the preoptic area, pattern 2 was observed for 5-HIAA; pattern 3 for dopamine and adrenaline. The frontal cortex and the hypothalamus, including the paraventricular nucleus (PVN), showed consistent patterns. Other brain regions, such as the thalamus or the dorsal raphe, have only been investigated in one or two studies. In the thalamus, 5-HT levels seemed to follow pattern 1, while noradrenaline levels seemed to follow pattern 3. In the dorsal raphe, 5-HIAA levels seemed to follow pattern 1.

Besides brain region, other factors seemed to modify monoamine levels, e.g. the age of the animals [47], genetic factors [48,49], the environment [47] or the sex of the animals [50]. Furthermore, in female rats, the menstrual cycle also seemed to play a role [51].

Monoamine levels seemed to decrease or to lose rhythmicity with age; in older animals levels were lower than in younger animals [47]. Comparing studies with similar characteristics apart from the age of the animals, older animals (22–27 months) showed stable dopamine levels in the striatum, while younger animals (1–6 months) followed the fluctuating pattern 1 [47,52,53].

#### Monoamine measurements and naturally occurring sleep stages

The patterns in monoamine concentrations during naturally occurring sleep stages are described by monoamine in Tables 7, 8, 9, 10, 11.

Monoamine levels fluctuate between wakefulness and naturally occurring sleep stages (SWS and REM). Like in the preceding section, we describe the findings with 3 general patterns; pattern A: monoamine levels decrease from wakefulness to SWS and decrease further to REM; pattern B: monoamine levels increase from wakefulness to SWS and increase further to REM; and pattern C: levels remain stable during wakefulness and both sleep stages. Some studies do not follow these general patterns, as described below.

All monoamines have been shown to fluctuate according to pattern A in at least one brain region, except for adrenaline, which was not studied within our sample. Serotonin levels match pattern A in 11 of the 15 reported regions (PVN/VMN, amygdala, locus coeruleus, preoptic area, hippocampus, PPT, medial reticular pontine formation, cortex, thalamus, gigantocellular reticular nucleus, caudate nucleus). Cortex noradrenaline levels, and prefrontal/frontal cortex dopamine levels also followed Pattern A.

The patterns may again differ by brain region; for some regions a specific pattern was observed, while for others, patterns varied. For instance, noradrenaline and serotonin levels followed pattern A in the amygdala and locus coeruleus, while dopamine levels followed pattern C. Similarly, DOPAC, 5-HIAA, and noradrenaline levels followed pattern A in the PVN/VMN, while, DOPAC levels followed pattern B.

Measurement characteristics and study designs of the included studies were heterogeneous, which could explain observed inconsistencies. For example, in the dorsal raphe, serotonin levels seemed to follow either pattern A or a pattern where levels are high during wakefulness and SWS, and become lower during REM.

Our meta-analyses showed that serotonin and noradrenaline levels overall followed pattern A; they decreased from wakefulness to SWS and decreased further to REM sleep (Figures 2 and 3).

For serotonin, concentrations during SWS and REM both showed significant decreases compared to wake; p < 0.0001 (95 Confidence Interval (CI) SWS [–0.31; –0.23], REM [–0.48; –0.39]; I^2^ = 98.4%). Our sensitivity analyses confirmed the findings from the NMA; the overall effect for SWS versus wakefulness was –1.45 (SMD) with CI 95% [–2.07; –0.82], p < 0.01 and I^2^ = 66%. The overall effect for wakefulness versus REM was –1.61 (SMD) with CI 95% [–2.36; –0.86], p < 0.01 and I^2^ = 68% (appendix 5–6).

For noradrenaline, the concentrations during SWS and REM also showed significant decreases compared to wake; p < 0.0001 (CI 95% SWS [–1.74; –0.69], REM: [–3.44; –2.12]; I^2^ = 99.5%). Our sensitivity analyses again confirmed these findings; the overall effect for SWS versus wakefulness was –1.54 (SMD) with CI 95% [–2.19; –0.89], p = 0.01 and I^2^ = 53%. The overall effect for wakefulness versus REM was –2.58 (SMD) with CI 95% [–4.48; –0.69], p < 0.01 and I^2^ = 80% (appendix 7–8).

#### Monoamine measurements and sleep deprivation

SD alters dialysate monoamine concentrations. Most SD studies measured serotonin levels. Monoamine levels mainly increased during and/or after SD, except for serotonin which has been shown to both increase and decrease (Table 12, 13, 14, 15, 16, 17).

The increases in monoamine levels during SD seemed reversible. For instance, DOPAC and 5-HIAA levels in the basal forebrain, and 5-HT levels in SCN increased during SD but returned to baseline during recovery. However, recovery was not always observed; for instance, in the nucleus accumbens, dopamine, noradrenaline, and adrenaline levels all increased and in the posterior hippocampus serotonin levels remained elevated after SD. Besides, in the gigantocellular reticular nucleus, serotonin levels dropped with a factor 100 during SD, and they remained decreased during recovery (128). Similar patterns were observed for serotonin levels in the frontal cortex and in the hippocampus, albeit with a lower amplitude (115). The SD-induced changes in the hippocampus could be specific to parts of this brain region. Serotonin levels were observed to decrease during SD in the ventral hippocampus, while increases were observed in the posterior hippocampus. Findings during recovery were inconsistent; both a decrease and an increase compared to baseline were observed for serotonin (80, 133).

### Medial prefrontal cortex experimental data

Monoamines levels remained fairly stable over baseline, as well as during SD and subsequent recovery (Table 18). Differences between the stages were not significant (p ≥ 0.07), except for DOPAC; χ^2^(3) = 8.486, p = 0.037 (Figure 4). The subsequent post-hoc tests showed a significant difference only between baseline light and dark, with a decrease in DOPAC levels from the light phase to the dark phase: T = 0, p = 0.018. The SD and recovery periods were not different from baseline (for SD versus light: T = 4, p = 0.091, for recovery versus light: T = 15, p = 0.886)

## Discussion

This systematic review provides a full overview of the available evidence on monoamine levels in brain microdialysates in relation to CRs and sleep. To the best of our knowledge, this is the first systematic review on this subject. It includes all relevant studies retrieved by searches in two important databases. We are also the first to implement a network meta-analysis for the direct comparison of three sleep-wake stages. Other systematic reviews on sleep in animals have focussed on adenosine [137] and anxiety-related behaviour [39].

In this review, we showed that monoamine levels fluctuate with CRs and naturally occurring sleep stages. In line with their function as “arousal” transmitters, they generally decrease from wakefulness to SWS, and further decrease from SWS to REM [138]. For noradrenaline and serotonin, we confirmed this with meta-analyses.

Monoamines are thought to promote wakefulness via a network comprising the brainstem, thalamus, hypothalamus, basal forebrain, and cortex. The brainstem contains several wake-promoting nuclei: the locus coeruleus (noradrenaline), the dorsal and median raphe nuclei (serotonin), the ventral periaqueductal grey, the substantia nigra and the ventral tegmental area (dopamine). More specifically, monoamines were thought to inhibit sleep-promoting regions such as the ventrolateral optic area (VLPO) [15,16]. Recent evidence suggest that the monoaminergic pathways may not cause sleep promotion, but counteract unpredicted shifts in CRs or effects of stressors [139].

While our systematic review focusses on monoamines, these neuromodulators do not act in isolation. For instance, the SCN provides input to the above-mentioned brainstem nuclei to synchronize sleep-wake regulation with the environmental light-dark cycle. Glutamate and acetylcholine release in the SCN depends on input from the laterodorsal and pedunculopontine tegmental nuclei [140], and the SCN receives cholinergic input from the basal forebrain [141], which seem involved in phase-shifting activity patterns in response to changing light-dark rhythms.

We exclusively addressed the release of monoaminergic neurotransmitters. Neurotransmitters exert their actions via binding to receptors. It is important to also analyze patterns in the expression of these receptors. Circadian variations in receptor expression have been shown for e.g. adrenergic, muscarinergic, opioidergic, gabaergic, and dopaminergic receptors [142]. Besides, nicotinergic receptors seem to be involved in regulation of the sleep cycle [143]. A recent narrative review on the neurochemistry of wake and sleep regulation can put our findings into further perspective [144].

Our review shows that while monoamine fluctuations differed between brain regions and monoamines, overall the monoamine concentrations seem to be higher during the active dark phase than during the inactive light phase. However, fluctuations also vary with factors such as sex, age, BMI, genetic status, temperature, season, and humidity [145,146,147]. Several mechanisms could be involved, and evidence is present for sleep-related changes in monoamine synthesis [148,149], degradation [150,151,152,153,154], receptors and transporters [148,155] and binding [156,157].

The conclusions we can draw are limited by the overall amount of evidence; the number of studies per condition is low. Mainly SD studies and adrenaline studies are currently underrepresented in the literature (only 15 studies for SD and 2 studies for adrenaline). New literature has probably appeared since we performed our search in September 2017. As systematic reviews generally take over a year from start to completion [158], a lag time from search to publication is hard to avoid. We do not expect the relative and absolute number of additionally available studies since September 2017 to change our conclusions. At this stage; further primary studies are still warranted. An update of this SR in a few years from now should be more conclusive. Current systematic review efforts should first focus on e.g. cholinergic neurotransmission and on receptor expression in relation to circadian rhythms, sleep, and sleep deprivation.

Overall conclusions on monoamine neurochemistry in relation to sleep and wake are further limited by the variations in experimental designs between the included studies. Heterogeneity was observed for e.g. species, group size, brain region, experimental duration, L/D cycle, type of SD (reviewed by [159]), flow rate, perfusate, probe membrane type and probe type.

The SD method itself could also affect monoamine concentrations, for example via stress. SD can be stressful for the animals because of e.g. social isolation, humidity, and/or restricted or forced locomotion. In SD studies, it is challenging to implement appropriate controls. While some SD techniques are considered less stressful (e.g. gentle handling) than others (e.g. disk over water), they all are intrusive and probably stressful when chronic.

Several studies have analysed SD-induced stress in animals [39,43,160,161,162]. The stress induced by our experimental SD method seems to be minimal; we previously showed that corticosterone concentrations were not elevated above the normal circadian peak [43]. In line with this, the currently presented data show stable levels of adrenaline, and noradrenaline.

The overall risk of bias for the studies included in our review is difficult to estimate because of poor reporting of experimental procedures. For example, reporting of power calculations and randomisation was mostly absent. Many details of the microdialysis technique were reported well; sample time, perfusion rate, matrix type, and analysis technique were reported in more than 96% of the included studies, and probe length and membrane type were reported in more than 85%. However, reporting of recovery and verification of probe placement could have been better. The list of questions we developed for risk of bias assessment of microdialysis studies (appendix 4) seems to provide an adequate reflection of the technique-related modulating factors [163]. We recommend its use for future systematic reviews on the microdialysis technique.

We compared the noradrenaline and serotonin concentrations between the 3 sleep-wake stages usually distinguished in rodents (wake, SWS and REM) with NMAs. Only noradrenaline and serotonin were analysed, as the total number of studies of other monoamines was low, and the heterogeneity between study designs was considered too high for meta-analyses in general. The results of our NMAs were consistent with pairwise comparisons of sleep stages with classic meta-analysis techniques. NMAs have previously been used for clinical trials; to compare treatment effects for more than two treatments, and even to rank a series of treatments in efficacy without direct comparisons being available [164]. The NMA technique seems well-suited for systematic reviews comparing several sleep-wake stages; it allows for multiple comparisons and missing data, while taking variations at the study-level into account.

Our primary data suggest that monoamine levels in the medial prefrontal cortex (mPFC) are stable during and after SD. The mPFC is involved in several sleep-dependent processes such as attention, memory, incentive processing, decision making, and emotional regulation, which rely, at least partially, on the monoaminergic pathways [17,18,19,165,166]. The frontal cortex, and notably the mPFC, deactivate during sleep and SD [167,168]. Probably, other neurotransmitters and neuromodulators than the studied monoamines are involved.

Our systematic review provides a complete overview of the previously published SD- and CR-related monoamine data in the mPFC. Concerning CR, in rats, 7 CRs studies show that monoamine levels fluctuated with CRs. Dopamine, noradrenaline, 5-HT, and 5-HIAA levels augmented during the dark phase and decreased or remained stable during light phase. DOPAC levels were higher during the dark phase than during the light phase. Concerning SD, only 1 SD study in mice showed that noradrenaline levels increased during SD.

In contrast to these preceding studies, our primary data show no variation in SD- and CR-related mPFC monoamines, except for DOPAC levels, which were higher during baseline light than during baseline dark. The variability of our data is high, probably due to occasional blockages of the microdialysis set-up. However, in the same samples, we did find normal circadian curves and minimal effects of SD for corticosterone [43]. We used non-parametric analyses of the median values per 12h-phase to prevent the variability affecting our outcomes. The differences between the findings of preceding studies and our data could be caused by differences in experimental design (e.g. method of SD, probe size, flow rate, precise coordinates and probe angle). At this stage, the overall effects of CR and SD on mPFC monoamine levels remains unclear.

Sleep is required for several fundamental physiological processes, extending beyond the monoaminergic pathways. This paper shows that monoamines fluctuate with CRs, sleep stages, and SD. The monoamines are affected by several factors including e.g. brain region, species, sex, age, BMI, genetic status, temperature, season and humidity. Monoamines may not be part of the basic mechanism underlying the regulation of the sleep-wake cycle [134]. However, their involvement in sleep-wake regulation seems clear. Primary studies are still warranted to clarify how.

## Additional Files

The additional files for this article can be found as follows:

10.5334/jcr.174.s1 Appendix 1. Study characteristics of included publications on circadian rhythms.

10.5334/jcr.174.s1 Appendix 2. Study characteristics of included publications on sleep stages.

10.5334/jcr.174.s1 Appendix 3. Study characteristic of included publications on sleep deprivation.

10.5334/jcr.174.s1 Appendix 4. Risk of bias – questions adapted to microdialysis studies.

10.5334/jcr.174.s1 Appendix 5. Forest plot comparing serotonin concentration (nanomole/L) during wake and slow waves sleep.

10.5334/jcr.174.s1 Appendix 6. Forest plot comparing serotonin concentration (nanomole/L) during wake and rapid-eye movement sleep.

10.5334/jcr.174.s1 Appendix 7. Forest plot comparing noradrenaline concentration (nanomole/L) during wake and slow waves sleep.

10.5334/jcr.174.s1 Appendix 8. Forest plot comparing noradrenaline concentration (nanomole/L) during wake and rapid-eye movement sleep.
