# Supplementary material for: Brain Microdialysate Monoamines in Relation to Circadian Rhythms, Sleep, and Sleep Deprivation – a Systematic Review, Network Meta-analysis, and New Primary Data
Source: J Circadian Rhythms. 2019 Jan 14;17:1. doi: 10.5334/jcr.174 (PMC6337052; doi:10.5334/jcr.174)

Appendix 1: Study characteristics of included publications on circadian rhythms

Reference_ID	N	Strain	Age / Weight	Sex (M/F/Both)	Lights on at (hours)	Flow rate (µL/min)	Probe length (mm)	Membrane/Probe type	Dialysis matrix	Wash-out time (hours)	Sample time bin (min)	Sample analysis
Alfinito et al (2009)	3-5	Sprague-Dawley	2-3 months	F	?	1	2	CMA/12	aCSF	3	30	HPLC-ECD
Azekawa et al (1991)	6	Wistar	250-350g	M	7	2	1.5	5kDa cut off	Ringer	24	20	HPLC-ECD
Barassin et al (2002)	9	Wistar	250-300g	M	?	1	1.5	U shaped, kDa9 cut off	aCSF + 5µM clomipramine	3	60	HPLC-ECD
Barbier et al (2007)	3-6	Sprague-Dawley	300-350g	M	6	1	4	Eicom	aCSF	?	60	HPLC- CD
Castaneda et al (2004) A1,2	5 (DA) 7 (DOPAC)	Wistar	2-4 months; 250-350g	M	20	1.25	4	Homemade, 5000 Da cut off	aCSF	?	30	Reverse phase HPLC-ECD
Castaneda et al (2004) B1,2	5 (DA) 8 (DOPAC)	Wistar	2-4 months; 250-350g	M	20	1.25	2	Homemade, 5000 Da cut off	aCSF	?	30	Reverse phase HPLC-ECD
Decker et al (2005)	6	Sprague-Dawley	40 PND	M	7	1.5	3	CMA/12	aCSF	?	30	HPLC-ECD
De Marquez-Prado et al (2000)	4	Wistar	2-3 months ;250-350g	M	8	1.25	4	Concentric, home-made, 5kDa cut off	aCSF	3	60	Reverse phase HPLC-FD
Drijfhout et al (1996)	4	Albino	250-300g	M	6	1.8	0.22	Dialysis tube, saponified cellulose	Ringer	?	20	Online derivation HPLC-FD
Dudley et al (1998)A	12	Syrian	Adult	M	7	1.2 or 1.8	1	Hemicellulose dialysis tubing, 12 kDa cut off	aCSF	2	60	HPLC-ECD with radial flow
Dudley et al (1998)B	5	Syrian	Adult	M	7	1.2 or 1.8	1	Hemicellulose dialysis tubing, 12 kDa cut off	aCSF	2	60	HPLC-ECD with radial flow
Dugovic et al (2009)	3-11	Sprague-Dawley	300-350g	M	6	1	4	Eicom	aCSF	?	30	HPLC-ECD
Ezrokhi et al (2014)	8	spontaneously hypertensive & Wistar	16 weeks; 334 ± 4g	M	5	0.12	1	32-gauge dialysis probe, 20kDa cut off	Ringer	19	120	HPLC-ECD (radial flow cell)
Ferris et al (2014) A	?	Sprague-Dawley	375-400g	M	?	0.8	2	CMA/Microdialysis	aCSF	8	30	HPLC-ECD
Ferris et al (2014) B & C	?	DAT KO and wild type	28-35g	?	?	0.8	2	CMA/Microdialysis	aCSF	8	30	HPLC-ECD

Reference_ID	N	Strain	Age / Weight	Sex (M/F/Both)	Lights on at (hours)	Flow rate (µL/min)	Probe length (mm)	Membrane/Probe type	Dialysis matrix	Wash-out time (hours)	Sample time bin (min)	Sample analysis
Fetissov et al (2000)1,2	9	Lean Zucker	350-400g	M	6	1.2	1	CMA 12, 20 kDa cut off, polycarbonate membrane	Ringer type	4?	20	reverse phase HPLC-ECD
Garabette et al (2000)	4-8	Wistar	260-300g	M	7 or 19	1.2	2.2	Gambro membrane, 6 kDa cut off	aCSF + 1µM citalopram	18	15	reverse phase HPLC-ECD
Glass et al (1992) A and D	6	Siberian	8-15 weeks; 40g	M	8	1	1.5	Hemicellulose dialysis tubing; 6kDa cutoff	aCSF	?	60	HPLC-ECD (radial flow cell)
Glass et al (1992) B	1	Siberian	8-15 weeks; 40g	M	8	1	1.5	Hemicellulose dialysis tubing; 6kDa cutoff	aCSF	?	60	HPLC-ECD (radial flow cell)
Glass et al (1992) C	7	Siberian	8-15 weeks; 40g	M	8	1	1.5	Hemicellulose dialysis tubing; 6kDa cutoff	aCSF	?	60	HPLC-ECD (radial flow cell)
Glass et al (1993)a	7	Syrian	8-15 weeks; 125g	M	8	1	1	Hemicellulose dialysis tubing; 6kDa cutoff	aCSF	?	60	HPLC-ECD
Glass et al (1993)b	6	Syrian	?	M	7	1	0.8-1	Hemicellulose dialysis tubing; 6kDa cutoff	aCSF	?	60	HPLC-ECD
Glass et al (1993)c	?	Djungarian	>90 days	M	8	1	3	Nitrocellulose hollow fiber dialysis membrane, 6kDa cut toff	aCSF	?	60	reverse phase HPLC-ECD
Grossmann et al (2000)	6	Syrian	10-12 weeks	M	5	1.2	?	Hemicellulose, 12 kDa cutoff	aCSF + 4µM Fluoxetine	1	20	HPLC-ECD
Grossmann et al (2004)	4	Syrian	8-10 weeks	M	?	1.2	1	12kDa cut off	aCSF + 4µM fluoxetine	2	60	HPLC-ECD
Gonzalez-Pina et al (2003)	7	Wistar	280-300g	M	?	1	1	BAS	aCSF	?	60	HPLC-ECD
Hucke et al (1998)1	2	Wistar	200-230g	F	6	1.3	4	CMA/11	?	?	20	Reverse phase HPLC-ECD
Hucke et al (1998)2	2	Wistar	200-230g	F	6	1.3	4	CMA/11	?	?	20	Reverse phase HPLC-ECD
Hood et al (2010)	?	Wistar	300g	M	8	1	2.5	Custom made, Fisher Scientific, 13kDa	aCSF	6	30	HPLC
Huang et al (2008)	1	Sprague-Dawleys	220-250g	M	6	2	?	TC-4	aCSF	?	10	FD-HPLC online

Reference_ID	N	Strain	Age / Weight	Sex (M/F/Both)	Lights on at (hours)	Flow rate (µL/min)	Probe length (mm)	Membrane/Probe type	Dialysis matrix	Wash-out time (hours)	Sample time bin (min)	Sample analysis
Izumo et al (2012)	7-8	Wistar	8 weeks; 180-200g	F	7	1	?	?	Ringer	2	20	HPLC-ECD
Jitsuki et al (2009) A	7	Wistar–Imamichi	2-3 months	M	5	1.2	1	AI-4-1	aCSF	?	30	HPLC-ECD
Jitsuki et al (2009) B & C	10	Wistar–Imamichi	2-3 months	F	5	1.2	1	AI-4-1	aCSF	?	30	HPLC-ECD
Kalen et al (1989)	4(NAD, 5-HT, 5-HIAA)<br>2 (NAD)	Sprague-Dawley	300g	F	?	2	8	Flexible SCE cellulose tubing , 10kDa cut off	Ringer +1 µM indalpine for 3 rats	0.5	30	HPLC-FD
Knoch et al (2004)	?	Syrian	Adult	M	?	1.2	1	Hemicellulose dialysis tubing, 12kDa cut off	aCSF + 4µM citalopram	?	60	HPLC-ECD
Linthorst et al (1994)	6-7	Wistar	250g	M	7:30	2	4	CMA/12, 20kDA cut off	Sterile pyrogen free Ringer	?	30	HPLC-ECD
Liu et al (2005)	1	Sprague-Dawley	?	M	11	2	4	CMA/12, 20kDA cut off	aCSF	?	10	HPLC-FD
Liu et al (2006) A	1	Lewis	2-4 months	M	6	2	4	CMA/12, 20 kDa cut off	aCSF	?	10	HPLC-FD
Liu et al (2006) B	6	Sprague-Dawley	2-4 months	M	6	2	4	CMA/12, 20 kDa cut off	aCSF	?	10	HPLC-FD
Liu et al (2006) C	8	Outbred wistar, per1-luc TG	2-4 months	M	6	2	4	CMA/12, 20 kDa cut off	aCSF	?	10	HPLC-FD
Liu et al (2006) D	4	PVG	2-4 months	M	6	2	4	CMA/12, 20 kDa cutoff	aCSF	?	10	HPLC-FD
Liu et al (2006) E	4	Lewis	2-4 months	M	6	2	4	CMA/12, 20 kDa cutoff	aCSF	?	10	HPLC-FD
Liu et al (2006) F	4	Syrian	2-4 months	M	6	2	4	CMA/12, 20 kDa cutoff	aCSF	?	10	HPLC-FD
Lopez-Rodriguez et al (2003) a	5	Sprague-Dawley	300-500g	M	1	2	4	CMA/11	aCSF	?	90	HPLC-ECD
Luo et al (1998)	5-6	Syrian	14 weeks; 208± 5g	M	8.5	0.12	1	32-gauge dialysis probe, 20kDa cut off	Filtered Ringer's solution	5	60	HPLC-ECD (radial flow cell)
Luo et al (1999)	5-6	Syrian	14 weeks; 185-195g	M	8.5	0.12	1	32-gauge dialysis probe, 20kDa cut off	Filtered Ringer's solution	3	120	HPLC-ECD
Luo et al (2000)	5-6	Syrian	14 weeks; 185± 10g	M	0	0.12	?	?	Ringer	?	17	HPLC-ECD (radial flow cell)
Luo et al (2004)	10	?	?	?	?	?	?	?	?	?	24	HPLC

Reference_ID	N	Strain	Age / Weight	Sex (M/F/Both)	Lights on at (hours)	Flow rate (µL/min)	Probe length (mm)	Membrane/Probe type	Dialysis matrix	Wash-out time (hours)	Sample time bin (min)	Sample analysis
Morien et al (1995)1 and 2	6	Albino Sprague-Dawley	350-400g	M	7	2	2	Cellulose fiber spectrum,membrane 6kDa cut off	Ringer	?	20	Reverse phase HPLC-ECD
Mitome et al (1994)a	7	Wistar	12 weeks; 450-550g	M	6	1	1	1kDa cut off	"Collagen solution"	?	120	HPLC-ECD
Mitome et al (1994)b	7	Wistar	?	M	6	1	0.6[2]	Homemade, 1kDa cut off	Ringer	2	120	HPLC-ECD
Nakayama et al (1993)	4	Wistar	300-350g	M	8	?	3	Dialysis probe	?	12	23	?
Nakayama et al (2002) 1,2, and 3	6	Wistar	300-350g	M	8	2	3	50kDa cut off	Ringer	?	23	HPLC-ECD
Oshima et al (2003)*	6 (5-HT) 7(5-HIAA)	C57Bl/6N	12-16 weeks	M	6	2	3	CMA/11,cuprophane, 6kDa cut off	Ringer	?	30 then 60	HPLC-ECD
Paulson et al (1994)1	?	Holtzman	Adult; 250-300g	M	6	1.5	3	Concentric-style probes	Perfusion medium	?	20	HPLC-ECD
Paulson et al (1994)2	?	Holtzman	Adult; 250-300g	M	6	1.5	2	Concentric-style probes	Perfusion medium	?	20	HPLC-ECD
Paulson et al (1996)1	17	Han-Wistar	Adult; 250-300g	M	6	1.5	2	Coated cyanoacrylate glue membrane	Perfusion medium	?	20	HPLC-ECD
Paulson et al (1996)2	18	Han-Wistar	Adult; 250-300g	M	6	1.5	2	Coated cyanoacrylate glue membrane	Perfusion medium	?	20	HPLC-ECD
Pealva et al (2002)A,B,C	7-13	CHR-R1 +/+, +/-, -/-	12-16 weeks;38g	M	6	2	3	CMA/11,cuprophane, 11kDa cut off	Sterile, pyrogen-free Ringer	?	30	HPLC-ECD
Robinson et al (1991)	7	?	Adult	F	?	2	5	CMA/10, 20kDa cut off	Ringer	2-3	20	HPLC-ECD
Sano et al (1992)A, C	?	Wistar	2-6 months	M	6	1.5	3.2	5kDa cut off	Ringer	3	30	HPLC-ECD
Sano et al (1992)B	?	Wistar	22-27 months	M	6	1.5	3.2	5kDa cut off	Ringer	3	30	HPLC-ECD
Sano et al (1992)D	?	Wistar	24-26 months	M	6	1.5	3.2	5kDa cut off	Ringer	3	30	HPLC-ECD

Reference_ID	N	Strain	Age / Weight	Sex (M/F/Both)	Lights on at (hours)	Flow rate (µL/min)	Probe length (mm)	Membrane/Probe type	Dialysis matrix	Wash-out time (hours)	Sample time bin (min)	Sample analysis
Sayer et al (1999)	4	Wistar	240-260g	M	6	1	2	CMA/12	aCSF + citalopram (1 mM)	1.5	15	HPLC-ECD
Smriga et al (2000)a	3-4	Wistar	250-280g	M	7	1	2	50kDa cut off	Modified Ringer	4	20	HPLC
Smriga et al (2000)b	3	Wistar	250-280g	M	7	1	1	50kDa cut off	Modified Ringer	4?	15	HPLC
Smriga et al (2002)	4	Wistar	250-280g	M	7	1	2	50kDa cut off	Modified Ringer	?	30	HPLC
Smith et al (1992)	5	Wistar	275-300g	M	7	0.057	4	Homemade probe (cellulose tubing)	aCSF	7	90	HPLC
Stanley et al (1989)a	11	Sprague-Dawley	Adult	M	9	1	2	6kDa cut off	Ringer	24	20	Reverse Phase HPLC-ECD
Stanley et al (1989)b	11	Albino Sprague-Dawley	360-380g	M	9	1	5	Homemade 6 kDa cut off	Ringer	?	120	Reverse Phase HPLC-ECD
Sun et al (2002)	1-5	Sprague-Dawley	Adult; 200-300g	M	11	2	3-4	CMA/12, 20kDa cut off	aCSF	?	10	HPLC-FD
Sun et al (2003)	113; 1 representative	Sprague-Dawley	220-225g	M	11	2	4	CMA/12	aCSF	132	10	HPLC-FD
Takahashi et al (1998)	5-10	Wistar	200-250g	?	?	2	3	50kDa cut off	Physiological solution	?	20	Reverse Phase HPLC-ECD
Verhagen et al (2009)	7	Wistar	155-165g	F	2	2	4	U-shaped tip, dialysis fiber, 10kDa cut off	Ringer	6	60	HPLC-ECD
Yang et al (2013)A,B,C	14	SERT +/+, +/-, -/-	?	Both	4	3	2	CMA/7	aCSF	3	3	HPLC-ECD

*Lower case letters indicate publications from the same authors and year; upper case letters represent separate groups within publications; numbers represent separate brain regions within animals *30 min samples 9h-20h and 60min samples 20h-9h.*

Abbreviations: M: Male; F: Female; DAT KO: Dopamine transporter Knock Out; CHR-R1: Corticopin release hormone receptor 1; SERT: serotonin transporter; aCSF: artificial Cerebrospinal Fluid; HPLC: High Pressure Liquid Chromatographic; ECD: Electrochemical Detection; FD: Fluorescence Detection; CD: Coulometric detection; µM: micromol/L; mM: milimol/L; kDa: KiloDalton.

Appendix 2: Study characteristics of included publications on sleep stages

Reference_ID	N	Strain	Age/Weight	Sex (M/F/Both)	Light at (hours)	Sleep detection	Flow rate (µL/min)	Probe length (mm)	Membrane/Probe type	Dialysis matrix	Washout time (hours)	Measurement duration (hours)	Baseline duration (hours)	Sample bin (min)	Sample analysis
Bellesi et al (2016)A1,A2	8	C57BL/6N	9-10 weeks	?	8	EEG and EGM	1	1.5	AN69, 12 kDa cut off	aECF	2	8	2	30	Reverse Phase Ion Paired HPLC-CD
Bjorvatn et al (2002) A1	8	Sprague-Dawley	250-300g	M	6	Fronto-frontal EEG, fronto-parietal EEG and neck muscle EMG	0.8	4	CMA/12	aCSF	1	7	0.66-1	20 every hour	HPLC-ECD
Bjorvatn et al (2002) A2	6	Sprague-Dawley	250-300g	M	6	Fronto-frontal EEG, fronto-parietal EEG and neck muscle EMG	0.8	3	CMA/12	aCSF	1	7	0.66-1	20 every h	HPLC-ECD
Blanco-centurion et al (2001) A	5	Wistar	250-300g	M	8	EOG and EMG	2.5	1	Microdialysis probe (Bioanalytical Systems)	aCSF	8	4	?	2	HPLC-ECD
De saint Hilaire et al (2000)	5	Wistar	250-300g	M	6	EEG, EMG	2	2	CMA microdialysis	aCSF	4	4	No BL	3	HPLC-ECD
Fiske et al (2006)1	11	Sprague-Dawley	200-350g	M	6	EEG and EMG	0.8	1	CMA /12	aCSF	2	10	?	10	HPLC-ECD
Fiske et al (2006)2	11	Sprague-Dawley	200-350g	M	6	EEG and EMG	0.8	3	CMA /12	aCSF	2	10	?	10	HPLC-ECD
Fiske et al (2008)1	6	Sprague-Dawley	250-300g	?	6	EEG and EMG	1	1	?	aCSF	2	?	?	5 at least	HPLC-ECD
Fiske et al (2008)2	6	Sprague-Dawley	250-300g	?	6	EEG and EMG	1	3	?	aCSF	2	?	?	5 at least	HPLC-ECD
Gronli et al (2007)	4	Sprague-Dawley	11 weeks ; 300g	M	7	EEG, EMG	1.2	4	CMA/12	aCSF	2	3-6	?	20	HPLC-ECD

Iwakiri et al (1993)	5	?	2.4-3.4kg	Both	?	EEG, EMG, EOG and PGO	1	3	4.5kDa cut off	Ringer	?	?	?	20	Reverse Phase HPLC-ECD
Lapierre et al (2012)	4	Nothern fur	Juvenile	?	?	EEG, EOG, neck EMG	?	?	?	?	?	?	?	?	HPLC-ECD
Lapierre et al (2013)a	4	Nothern fur	2-3 years; 20-21kg	Both	8	EEG, EMG, EOG	1.5	4	CMA/12 Elite Microdialysis Probe, 20kDa cut off	aCSF	?	40	At least 4	10	HPLC-ECD
Lapierre et al (2013)b	3	Nothern fur, Callorhinus ursinus	Juvenile	?	?	EEG, EOG and neck EMG	?	?	14 probes	?	?	?	?	?	HPLC-ECD
Lena et al (2005)1	6	Wistar	280-320g	M	8	EEG and EMG	1	3	CMA/12,20 kDa cut-off	Ringer	4	?	?	2	Capillary Electrophoresis
Lena et al (2005)2	6	Wistar	280-320g	M	8	EEG and EMG	1	2	CMA/12,20 kDa cut-off	Ringer	4	?	?	2	Capillary Electrophoresis
Lyamin et al (2016)	4	Nothern fur	2-3 years; 18-25kg	Both	8	EEG, EOG, EMG	1	4	CMA-12 Elite Microdialysis Probe, 20kDa cut off	aCSF	4	42	?	10	HPLC-ECD
Mukaida et al (2007)	6-7	Wistar	230-300g	M	7	EEG	2	2	A-I-8	aCSF	2	?	2	10	HPLC-ECD
Nicolaidis et al (2001)1,2	?	Wistar	250-300g	M	?	EEG + visual inspection	2	2	CMA, 20kDa cut off	Ringer like	3	?	?	6	Reverse phase HPLC-ECD
Orosco et al (1995)	5	Wistar	250-300g	M	6	EEG	2	2	Carnegie, 20 kDa cut off	Ringer	3	?	?	6	Reverse phase HPLC-ECD
Park et al (1999)	5	Sprague-Dawley	300-550g	M	7	EEG, EMG and dorsal hippocampus recording	2	4	CMA/11 or CMA/12	aCSF	4	72-96	?	5	HPLC-ECD
Park et al (2002)	5	Sprague-Dawley	300-580g	M	7	EEG, EMG and dorsal hippocampus recording	2	2	CMA/11	aCSF	24	72-96	?	5	HPLC-ECD
Penalva et al (2003) A	11	Wistar	220g	M	7:30	EEG and EMG	2	4	CMA/12	Sterile pyrogen-free Ringer	40-45	3	?	3	HPLC-ECD
Portas et al (1994)	6	?	3-5kg	?	N/A	EEG, EOG, EMG, PGO	3	2	CMA/10	aCSF	1.666	?	?	3	HPLC-ECD
Portas et al (1996)	5	?	3kg	M	N/A	EEG , EMG, EOG, PGO	1.5	1	CMA/10, 20 kDa cut off	aCSF	1 at least	10	120	5	HPLC-ECD
Portas et al (1998)1	7	Sprague-Dawley	240-295g	M	6	EEG, EMG	1	1	CMA/12	aCSF	?	8	180	5	HPLC-ECD

Portas et al (1998)2	4	Sprague-Dawley	240-295g	M	6	EEG, EMG	1	3	CMA/12	aCSF	?	8	180	5	HPLC-ECD
Python et al (2001)	6	Wistar	250-300g	M	8	EEG and EMG	2	2	20kDa cut off	Ringer type	4	3	?	2	Reversed Phase HPLC-ECD
Shouse et al (2000)a 1,2	8	Epilepsy model	Pre-adolescent	?	?	?	2	?	?	?	?	6-8	?	5	?
Shouse et al (2000)b 1,2	6	?	5-6 months; 2.1kg	Both	?	EEG, EOG, EMG	2	2	Polyamide tubing, Eicom DM-22 membrane	aCSF	?	At leas t 2	?	5	HPLC-ECD
Shouse et al (2001)a 1,2	8	?	6-6.5 months; 2.1kg	Both	?	EEG, EOG, EMG	2	2	Eicom DM-22	aCSF	?	1	1	5	HPLC-ECD
Shouse et al (2001)b 1,2	8	?	6-6.5 months; 2.1kg	Both	?	EEG, EOG, EMG	2	2	Eicom DM-22 membrane	aCSF	?	6-8	?	5	HPLC-ECD
Strecker et al (1999)	7-9	?	Adult	M	?	EEG, EOG,EMG, and PGO	1.5	2	CMA 10, 20 kDa cut off	aCSF	16	4	?	7.5-10	HPLC-ECD
Wilkinson et al(1991)	5	?	?	?	?	EEG,EMG, EOG	2	5	6kDa	Degassed dialysis solution	8	150	?	30	HPLC-ECD
Zeitzer et al (2002)	1	Homo sapiens	24 years old	M	?	EEG	0.5	?	?	aCSF	?	6.5	?	10	HPLC-ECD

Lower case letters indicate publications from same authors and year; upper case letters represent separate groups within publications; numbers represent separate brain regions within animals. Abbreviations: M: Male; F: Female EEG: Electroencephalogram; EMG: Electromyogram; EOG: Electrooculogram; PGO: Ponto-Geniculo-Occipital Wave Recording; aCSF: artificial Cerebrospinal Fluid; aECF: artificial Extracerebral Fluid; HPLC: High Pressure Liquid Chromatography; ECD: Electrochemical Detection; kDa: Kilo Dalton

Appendix 3: Study characteristic of included publications on sleep deprivation

Reference_ID	N	Strain	Age/Weight	Sex (M/F/Both)	Light at (hours)	Onset time SD (hours)	Flow rate (µL/min)	Probe length (mm)	Probe/Membrane type	Dialysate matrix	Washout time (hours)	Measurement duration (hours)	Baseline duration (hours)	Sample bin (min)	Sample analysis
Bellesi et al (2016)B1, B2	8	C57BL/6	9-10 weeks	?	8	17	1	1.5	AN69, 12 kDa cut off	aECF	2	8	2	30	Reverse Phase Ion Paired HPLC-CD
Bjorvatn et al (2002) B1	8	Sprague-Dawley	250-300g	M	6	7:30	0.8	4	CMA/12	aCSF	1	7	0.66-1	20	HPLC-ECD
Bjorvatn et al (2002)B2	8	Sprague-Dawley	250-300g	M	6	7:30	0.8	3	CMA/12	aCSF	1	7	0.66-1	20	HPLC-ECD
Blanco-centurion et al (2001)	7	Wistar	250-300g	M	8	20	0.5	1	Microdialysis probe (Bioanalytical Systems)	aCSF	16	48	?	≤ 3	HPLC-ECD
Grossmann et al (2000)B	12	Syrian	10-12weeks	M	5	13	1.2	?	Hemicellulose, 12 kDa cut off	aCSF + 4.0 µM Fluoxetine	1	11	3	20	HPLC-ECD
Grossmann et al (2000) C	6	Syrian	10-12weeks	M	5	13	1.2	?	Hemicellulose, 12 kDa cut off	aCSF + 4.0 µM Fluoxetine	1	11	3	20	HPLC-ECD
Lopez-Rodriguez et al (2003)a	6	Sprague-Dawley	300-500g	M	1	13	2	4	CMA/11	aCSF	?	24	24	1.5	HPLC-ECD
Lopez-Rodriguez et al (2003)b	6	Sprague-Dawley	300-350g	M	8	12	2	4	CMA/11	aCSF	?	16	3	30	HPLC-ECD
Murillo-Rodriguez et al (2016)	10	Wistar	250-300g	M	7	7	0.25	1	polyacrylonitrile, 30 kDa cut off	aCSF	24	6	?	20 every hour	HPLC-ECD

Penalva et al (2003) B	6	Wistar	220g	M	7:30	7:30	2	4	CMA/12	Sterile, pyrogen-free Ringer solution	40-45	6	3	3	HPLC-ECD
Penalva et al (2002) C	5	Wistar	220	M	7:30	7:30	2	4	CMA/12	Sterile, pyrogen-free Ringer solution	40-45	6	3	15	HPLC-ECD
Zant et al (2010)	?	Han-wistar	?	M	?	?	?	?	?	?	?	?	?	?	HPLC
Zant et al (2011)	8	Han-wistar	3-4 months; 300-400g	M	8:30	10:30	1	2	CMA/11	aCSF	?	10	1	20	HPLC-ECD

Lower case letters indicate publications from the same authors and year; upper case letters represent separate groups within publications; numbers represent separate brain regions within animals. Abbreviations: M: Male; aCSF: artificial Cerebrospinal Fluid; HPLC: High Pressure Liquid Chromatography; ECD: Electrochemical Detection; CD: Coulometric detection

Appendix 4: Risk of bias – questions adapted to microdialysis studies

Component	Comments/Clarification
1. Was any aspect of the experiment blinded?	Yes: Blinding was performed and adequately described for at least part of the experiment.<br>No: Blinding is not mentioned, and/or it was insufficiently described or performed
2. Was the location of probe implantation detailed by coordinates and verified after the experiments (histology or otherwise)?	Yes: The study described the coordinates used for implantation and histological or other verification confirmed the probe's location within the brain<br>No: At most the name of the brain region was given and/or verification of probe location was not mentioned.
3. Was the analysis technique described?	Yes: the measurement technique was described.<br>No: the measurement technique was not described
4. Was the probe recovery calculation reported? Are there corresponding values presented?	Yes: Probe recovery calculation is described. The value(s) is/are explicitly stated.<br>No: Neither are reported
5. Did the authors reported the timing and/or the phases of light/dark cycle?	Yes: Starting and finishing time of the experiment and the light/dark cycle are reported.<br>No: Timing and/or light/dark cycles were not reported.
6. If applicable: Is the technique used to determine sleep stages reported and is it valid?	Yes: Sleep stages were adequately determined.<br>No: sleep stage determination was either not reported or not adequate (non-validated algorithms, videotaping..)
7. If applicable: Was sleep deprivation properly performed and described?	Yes: sleep deprivation was adequately performed and described<br>No: Invalid method and/or insufficiently reported
8. Were the animal characteristics properly described?	Yes: The authors mentioned species, age, gender of the animals, and the number of animals used<br>No: The authors did not describe one or more of the above
9. Was a power analysis performed?	Yes: A proper power analysis was performed and reported<br>No: A power analysis was not reported
10. Was the study approved by an ethical board?	Yes/Not reported/ No
11. Is there a conflict of interest statement?	Yes/Not reported/ No

Appendix 5: Forest plot comparing serotonin concentration (nanomole/L) during wake and slow waves sleep

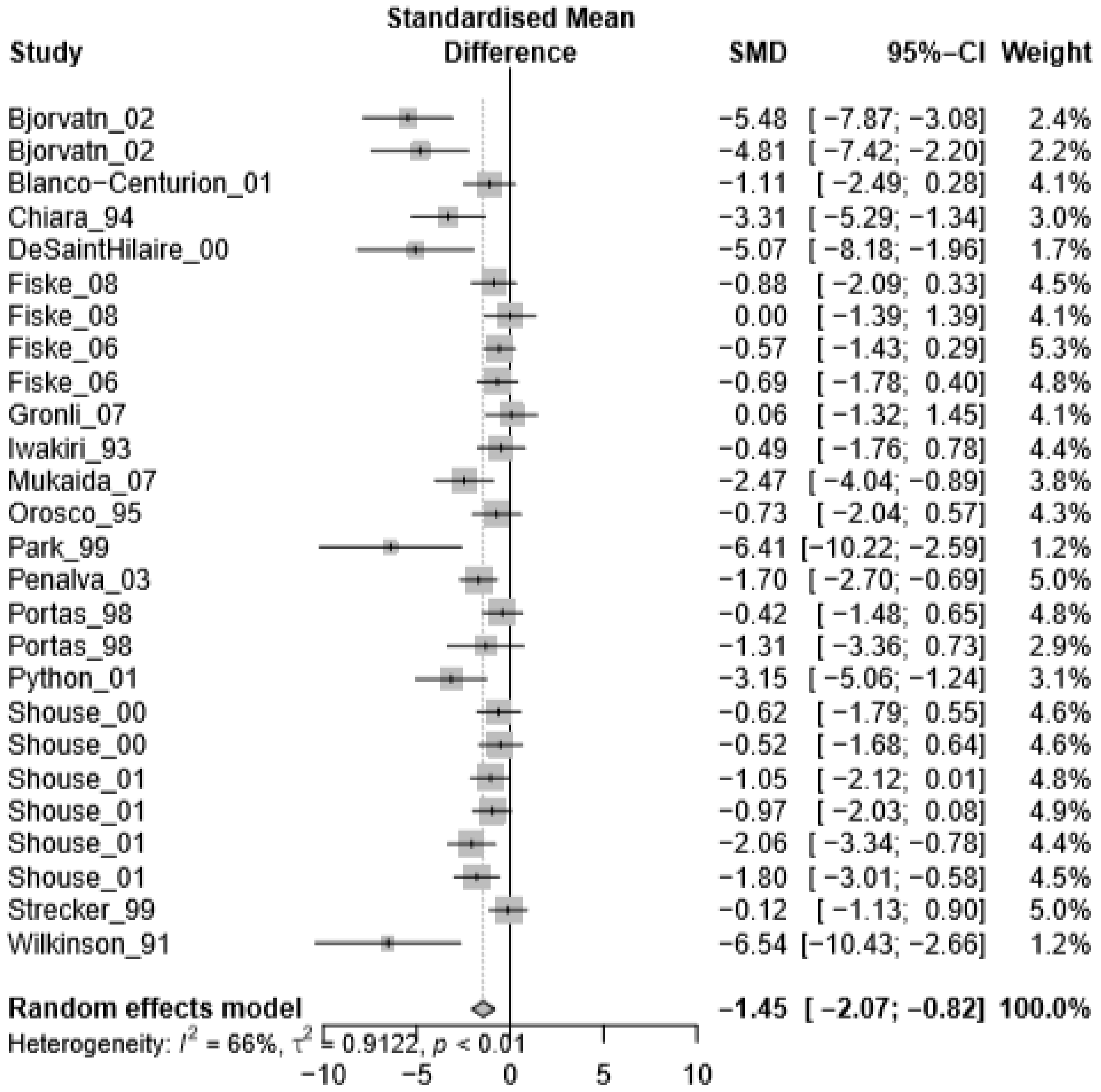

Appendix 6: Forest plot comparing serotonin concentration (nanomole/L) during wake and rapid-eye movement sleep

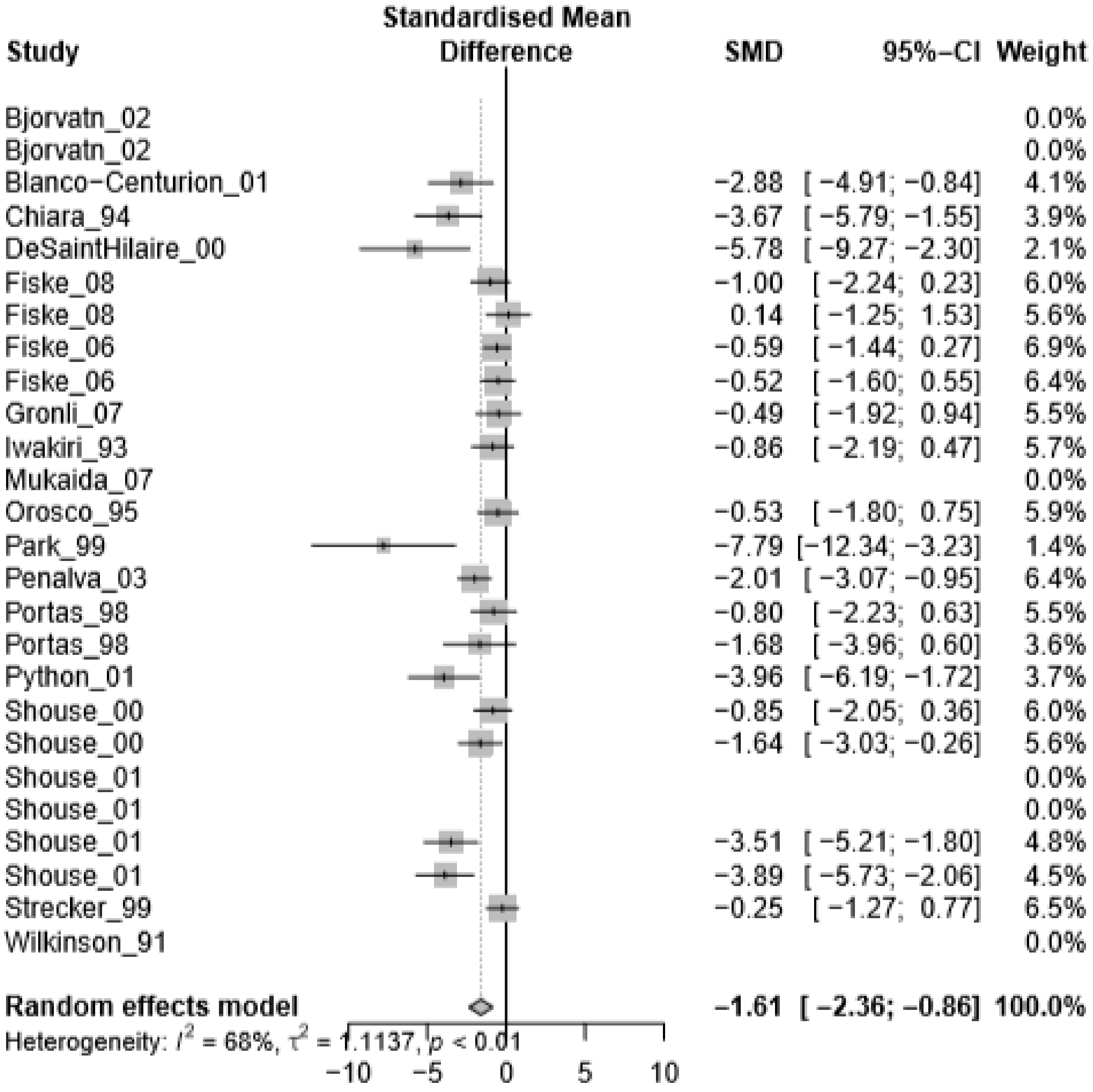

Appendix 7: Forest plot comparing noradrenaline concentration (nanomole/L) during wake and slow waves sleep

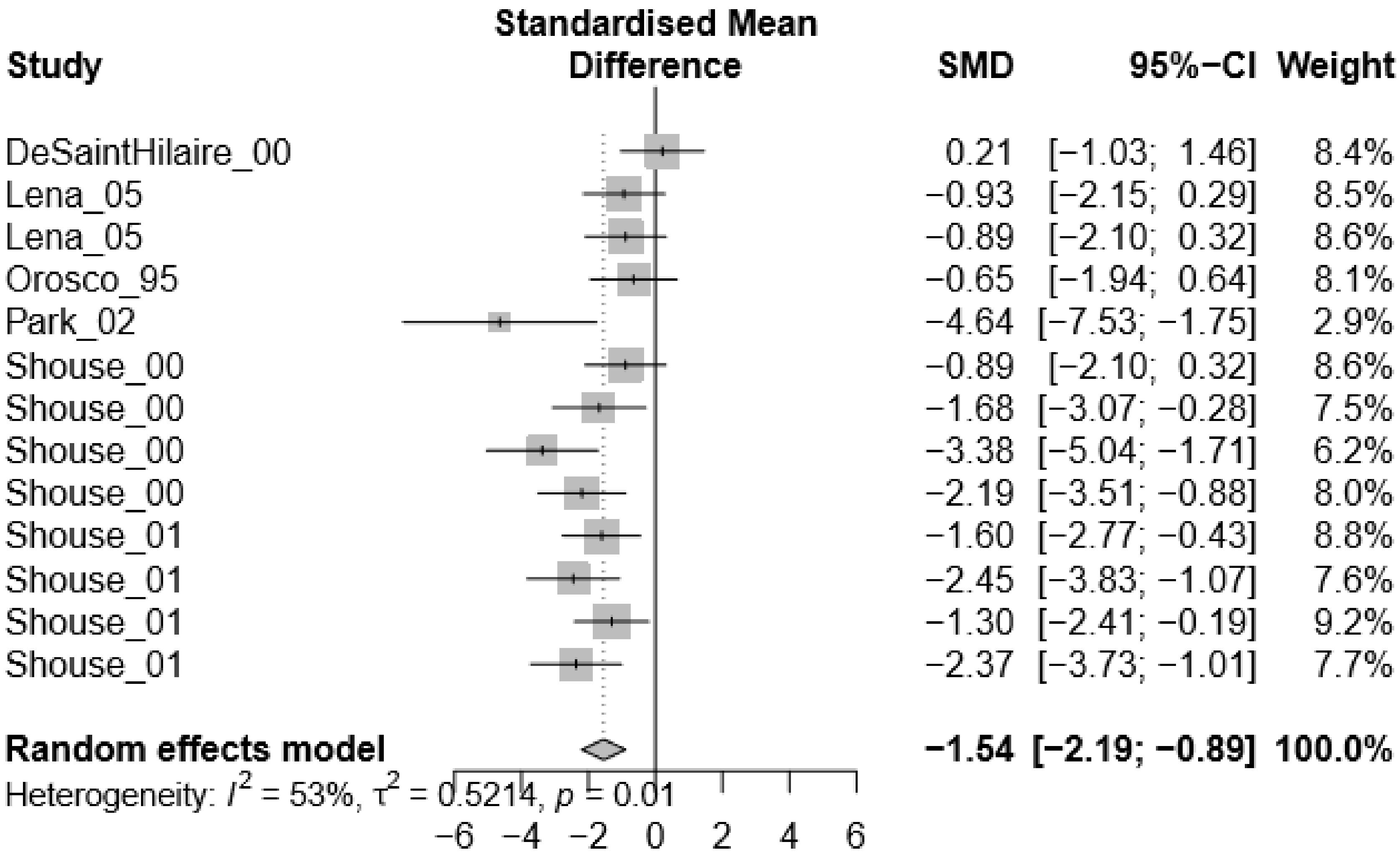

Appendix 8: Forest plot comparing noradrenaline concentration (nanomole/L) during wake and rapid-eye movement sleep

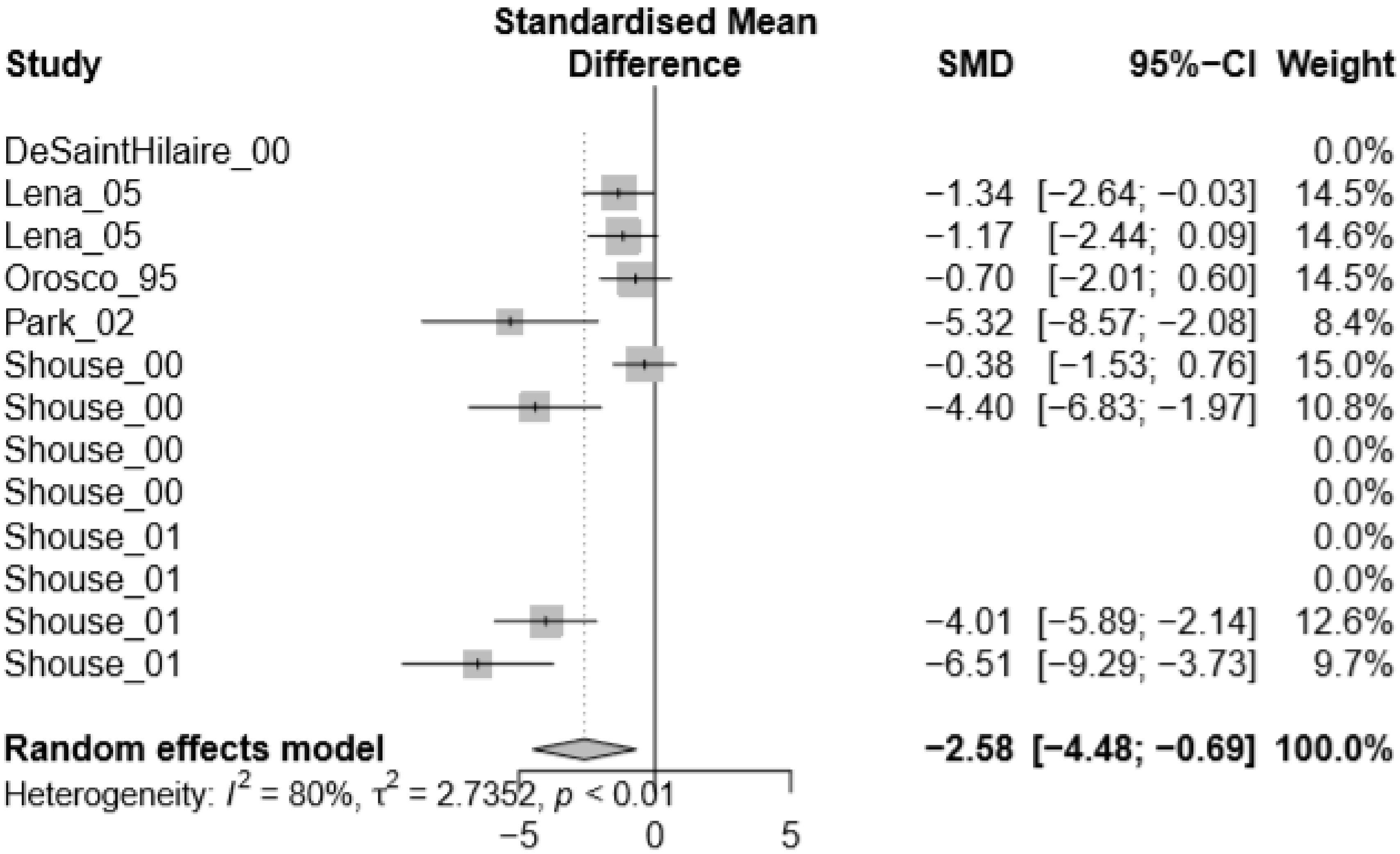

Supplement: Appendix 1. — Study characteristics of included publications on circadian rhythms. [file jcr-17-174-s1.pdf]
